# Discovery and characterization of a new genotype of *Salmonella enterica* serovar Bareilly isolated from diarrhea patients of food-borne outbreaks

**DOI:** 10.3389/fmicb.2022.1024189

**Published:** 2022-10-26

**Authors:** Nanjoo Park, Joon-Gi Kwon, Hongjun Na, Sohyun Lee, Ju-Hoon Lee, Sangryeol Ryu

**Affiliations:** ^1^Department of Food and Animal Biotechnology, Seoul National University, Seoul, South Korea; ^2^Department of Agricultural Biotechnology, Seoul National University, Seoul, South Korea; ^3^Research Institute of Agriculture and Life Sciences, Seoul National University, Seoul, South Korea; ^4^Center for Food and Bioconvergence, Seoul National University, Seoul, South Korea; ^5^Gyeonggi-do Research Institute of Health and Environment, Suwon, South Korea; ^6^Research and Development Center, Sanigen Co. Ltd., Anyang, South Korea

**Keywords:** *Salmonella* Bareilly, food-borne outbreak, whole genome sequence, genotyping, phylogeny

## Abstract

Since the first food-borne outbreak of *Salmonella enterica* serovar Bareilly in the UK (2010), it has been recognized as a new type of food-borne pathogen in *S. enterica.* To detect and characterize this new serovar pathogen in South Korea, a total of 175 *Salmonella* strains was isolated and 31 isolates were identified as *S.* Bareilly from various food-borne outbreaks between 2014 and 2018. While pulsed-field gel electrophoresis (PFGE) analysis using *Xba*I revealed two major groups (A and B) each with two subgroups (A1, A2/B1, B2), average nucleotide identity (ANI), single nucleotide polymorphism (SNP), and *in silico* multilocus sequence typing (MLST) analyses confirmed only two major groups. Interestingly, extended SNP analysis with 67 *S.* Bareilly strains from outbreaks in other countries revealed that A group strains between 2014 and 2016 shared a close evolutionary relationship with the strains from outside of South Korea; however, the B group strains in 2018 were located in a separate SNP tree branch. These findings suggest that the A group may share common ancestor with the strains of previous outbreaks in the UK or other countries, while the B group is a new genotype. Comparative virulence factor (VF) analysis between the A and B group strains showed that *S.* Bareilly in the B group has more various than that of the A group. A comparative biofilm formation assay supports for this, which B group strain GG-21 has higher biofilm formation activity than A group strain GG-07. Antibiotic susceptibility test of 31 *S.* Bareilly strains revealed high susceptibility to 17 tested antibiotics, suggesting that *S.* Bareilly can be easily treated by antibiotics.

## Introduction

*Salmonella enterica* is one of the most common food-borne pathogens, responsible for food-borne salmonellosis (Lamas et al., 2018). This species has more than 2,500 serovars including Typhimurium, Enteritidis, Typhi, Paratyphi, and Bareilly (Grimont and Weill, 2007). *S. enterica* is the primary food-borne pathogen causing salmonellosis with diarrhea, vomiting, and fever *via* consumption of the contaminated foods. *S. enterica* infections have been reported as the second most common single agent, with 896 outbreaks and 23,662 hospitalizations from 2009 to 2015 in the United States (Dewey-Mattia et al., 2018) and 6,340 hospitalizations from 2014 to 2018 in South Korea (Ministry of Food and Drug Safety [MFDS], 2018). While *S.* Typhimurium and *S.* Enteritidis are major salmonellosis-causing serovars, a new serovar bacterium, *S. enterica* serovar Bareilly, causing paratyphoid fever, was first identified in India in 1928; this bacterium belongs to the C1 serogroup with the antigenic formula 6,7,14: y: 1,5 (Bridges and Scott, 1931). In 2010, an *S.* Bareilly outbreak was reported in the UK, with 231 people infected by contaminated bean sprouts (Cleary et al., 2010). In the United States, an *S.* Bareilly outbreak was reported in 2012, resulting in 410 patients from 28 states and Washington DC to become infected after eating a raw scraped ground tuna product (Hoffmann et al., 2016). Since then, *S*. Bareilly is one of the most frequent serovars of enteric salmonellosis in the United States (Centers for Disease Control and Prevention [CDC], 2018; Dewey-Mattia et al., 2018). In addition, this serovar has been detected and has become one of the top 20 *Salmonella* serotypes for human disease-associated bacteria in the European Union/European Economic Area (EU/EEA) since 2016 (European Food Safety Authority and European Centre for Disease Prevention and Control [EFSA and ECDC], 2018). In particular, 25 *S*. Bareilly infection outbreaks were reported in the Czech Republic from 2013 to 2017 (Labská et al., 2021). Based on these previous outbreaks, *S.* Bareilly infections have widely spread in Western countries. Although *S.* Bareilly infection cases and outbreaks have been reported in the last decade, full pathogenic and taxonomical characterizations have not been conducted, and its prevalence has not been investigated in Eastern countries. Therefore, the pathogenesis, taxonomy, and prevalence should be further elucidated to understand and control this newly rising food-borne serovar of *Salmonella.*

To study the infection origin, taxonomy, and prevalence of *S.* Bareilly in the reported outbreaks, molecular biology and genomics-based analysis techniques including genomic DNA-based pulsed field gel electrophoresis (PFGE), house-keeping gene-based multilocus sequence typing (MLST), and whole genome sequence (WGS)-based single nucleotide polymorphism (SNP) were used. A 2010 UK outbreak study identified the *S.* Bareilly outbreak-associated PFGE profile (Cleary et al., 2010). Interestingly, this PFGE profile was also detected from a patient in Ireland who had visited London during the same period, suggesting infection with the same origin strain. Further investigations of the 2010 UK outbreak revealed that the origin of contamination was mung bean sprouts seeds imported from China or Myanmar (Cleary et al., 2010). A 2012 US outbreak study conducted outbreak-based PFGE analysis and WGS-based SNP analysis (Hoffmann et al., 2016). PFGE analysis showed that the PFGE *Xba*I pattern was indistinguishable from that of *S.* Bareilly isolates from a 2011 outbreak in Maryland. Furthermore, WGS-based SNP analysis revealed that *S.* Bareilly isolates from the 2012 outbreak were positioned on the same evolutionary branch as *S.* Bareilly isolates from the 2011 Maryland outbreak as well as frozen tuna scrape from India, suggesting that this *S.* Bareilly strain originated from a fishery facility in India (Hoffmann et al., 2016). The prevalence of *S.* Bareilly in commercial farms and retail markets was investigated in South Korea (Seo et al., 2018). A total of 45 isolates was obtained from egg products and related environmental samples, suggesting that *S.* Bareilly is predominant in chicken products and feces. PFGE pattern analysis showed two major patterns and two sub-patterns. However, resistance was observed in only 24.4% isolates against streptomycin and in 6.7% against cephalothin, indicating low antibiotic resistance activity of *S.* Bareilly. In recent years, advanced next-generation sequencing (NGS) technology has been available for comparative genome analysis (Pareek et al., 2011). With these NGS methods, genetic variation detection analyses using average nucleotide identity (ANI) and single nucleotide polymorphism (SNP) methods have enabled more precise phylogenetic characterization of isolated *S.* Bareilly at the genome level (Seeb et al., 2011). Therefore, to further clarify the origin of infection, taxonomical relationship, and prevalence of *S.* Bareilly, it was necessary to perform genome-level characterizations of isolates from food-borne outbreaks.

In this study, *S.* Bareilly isolates were obtained from South Korea patients with diarrhea, and serotyping was performed. Subsequent genome-level characterization using PFGE, ANI, SNP, and MLST analyses provided the evolutionary relationships and typical phylogenetic patterns in South Korea. Their whole genome sequences were analyzed to detect their virulence factors at the genetic level; antibiotic resistance and biofilm formation activities were investigated to confirm their phenotypic characterizations. Consequently, this study will be useful to extend our understanding of the evolutionary relationship and pathogenesis of *S.* Bareilly in South Korea and to provide basic information on control and regulation of pathogenic *S.* Bareilly for food safety.

## Materials and methods

### Sampling, isolation, and serotyping

Overall information on five food-poisoning outbreaks and their 26 patients and 5 canteen employees was listed in Table 1. Thirty-one rectal swab samples were collected from the patients and canteen employees (one sample per person) by standard rectal swab sample collection method in Gyeonggi-do, South Korea, by the Research Institute of Health and Environment from 2014 to 2018. For isolation of *S.* Bareilly, each sample was plated on *Salmonella-Shigella* (SS) agar (Oxoid, UK) and incubated at 37°C for 18–24 h. The isolates were identified using the VITEK 2 system with a commercial GN card (bioMerieux Inc., France). *Salmonella* serotyping was conducted according to the White-Kauffmann-Le Minor scheme, using somatic (O) provided by the Korea Disease Control and Prevention Agency (KDCA) and flagella (H) antisera purchased from Difco, USA.

**TABLE 1:** Summary of 31 *Salmonella* Bareilly outbreak strains isolated in Gyeonggi-do, South Korea from 2014 to 2018.

Case	Time	Strain	Origin	City	Venue	Symptom	Suspected food
I	September 2014	GG-01	Patient-1	Ansan	Cafeteria	Diarrhea	Steamed rice
		GG-02	Patient-2			Fever	Galbi-tang
		GG-03	Patient-3			Chill	Beef bulgogi
		GG-04	Patient-4				Mixed egg-jeon
		GG-05	Patient-5				Pork bulgogi
		GG-06	Patient-6				Fried vegetables
		GG-07	Patient-7				Pork ribs stew Sullung-tang Kimchi
II	August 2015	GG-08	Patient-8	Hwaseong	Cafeteria	Diarrhea	Jjajang-bap
		GG-09	Patient-9			Fever	Steamed rice
		GG-10	Patient-10			Abdominal pain	Pork cutlet
		GG-11	Patient-11				Stir-fried pork
		GG-12	Patient-12				Bellflower pickle
		GG-13	Patient-13				Kimchi
		GG-14	Patient-14				Soybean soup
		GG-15	Patient-15				Lettuce
		GG-16	Patient-16				
III	October 2016	GG-17	Employee-1	Yongin	Restaurant	Asymptomatic	Unknown
		GG-18	Employee-2				
		GG-19	Employee-3				
IV	October 2018	GG-20	Patient-17	Gimpo	Restaurant	Diarrhea	Kimbab
		GG-21	Patient-18			Fever	
		GG-22	Employee-4			Abdominal pain	
		GG-23	Employee-5			Asymptomatic	
V	October 2018	GG-24	Patient-19	Gunpo	Restaurant	Diarrhea	Kimbab
		GG-25	Patient-20			Fever	
		GG-26	Patient-21			Vomiting	
		GG-27	Patient-22				
		GG-28	Patient-23				
		GG-29	Patient-24				
		GG-30	Patient-25				
		GG-31	Patient-26				

### Pulsed field gel electrophoresis analysis

Pulsed field gel electrophoresis analysis of the *S*. Bareilly strains was performed according to the PulseNet protocol (Ribot et al., 2006). Agarose-embedded genomic DNA was digested with *Xba*I and separated by PFGE using a CHEF-Mapper XA system (Bio-Rad Laboratories, USA) at 6 V/cm for 18 h and 14°C with initial and final switch times of 2.16 and 54.17 s, respectively. The PFGE patterns were analyzed using BioNumerics software ver. 5.1 (Applied Maths, Belgium) with the Dice similarity coefficient of a 1.5% position tolerance.

### Whole genome sequencing

Genomic DNA of the isolates was extracted using an Intron G-spin™ Genomic DNA Extraction Kit (Intron Biotechnology, Korea). The integrity and concentration of DNA were determined by standard agarose gel electrophoresis and the Qubit 3.0 fluorometer (Thermo-Fisher Scientific, USA), respectively. Intact genomic DNA was sheared by a Covaris M220 sonicator (Covaris, USA), and the sequencing library was constructed using the Illumina TruSeq Nano DNA library prep kit with single-indexed adapters (Illumina, USA). The library was sequenced using the Illumina MiSeq platform with paired-end reads of 300 bp in length. The sequence quality of the raw data was assessed with FastQC software (Andrews, 2010). Low-quality sequence bases were trimmed using the Trimmomatic program version 0.33 (Bolger et al., 2014). Filtered and qualified read sequences were assembled using SPAdes version 3.13 (Bankevich et al., 2012).

### Bioinformatics analysis

*In silico* MLST profiling was performed using MLST version 2.0 on the CGE website (Larsen et al., 2012) with the following seven gene sequences: *aroC, dnaN, hemD, hisD, purE, sucA*, and *thrA*. Average nucleotide identity (ANI) values were calculated using JSpecies v1.2.1 based on the BLAST algorithm (Richter and Rosselló-Móra, 2009). The ANI-based phylogenetic tree was constructed with the calculated ANI values and visualized using RStudio software of R package (R Core Team, 2020). Virulence factors and antibiotic resistance genes were predicted using Virulence Factor Database (VFDB) (Liu et al., 2019) and Comprehensive Antibiotic Resistance Database (CARD) (Alcock et al., 2020). Pangenome analysis was performed using Roary Pangenome Pipeline (Page et al., 2015), and its visualization was conducted using the BLAST Atlas program in a GView server.^1^ For SNP determination, the qualified read sequences of whole genome sequences were mapped to *S*. Bareilly str. CFSAN000189 as reference genome sequence with bowtie2 program version 2.3.5.1 (Langmead and Salzberg, 2012), and variants were called using VarScan version 2.3 (Koboldt et al., 2009). The SNP patterns were compared with CFSAN SNP pipeline version 2.1.1 (Davis et al., 2015). Using the SNP comparison data, MEGA X (Kumar et al., 2018) was used to build a phylogenetic tree using the neighbor-joining method. Visualization of the phylogenetic tree was conducted with FigTree software (Excoffier et al., 2005).

### Selection of representative strains for comparative pangenome analysis

For efficient pangenome analysis, identical genotype strains among outbreak case groups were determined by ANI analysis with their WGS. When the ANI score had a 100% identity match, one strains from each group was randomly selected as a representative. Other strains with ANI scores that were not a 100% identity match were selected as individual representative strains.

### Invasion and adhesion assay

Invasion of *S.* Bareilly isolates into human intestinal epithelial Caco-2 cells (ATCC catalog No. HTB-37) was monitored using the gentamycin protection assay protocol as previously developed (Yoo et al., 2017). Before bacterial infection, 2.5 × 10^5^ Caco-2 cells/well were prepared in a 24-well cell culture plate (SPL Life Sciences Co., Korea). After incubation of *S*. Bareilly at 37°C for 4 h, Caco-2 cells were infected at a multiplicity of infection (MOI) of 10. After 30 min for infection, 100 μg/ml of gentamycin (Sigma, USA; final concentration) was added, and the infected cells were additionally incubated at 37°C for 1 h. The cells were washed three times with phosphate-buffered saline (PBS; Gibco, USA) and then lysed using 1% Triton X-100 (Sigma, USA). The lysed solution was serially diluted and plated onto Luria-Bertani agar (Difco, USA). The bacterial colonies were counted using a standard viable cell count to enumerate colony forming units (CFU). An adhesion assay of the *S.* Bareilly isolates was performed as previously described (Aviv et al., 2019) using cytochalasin D to prevent bacterial cell invasion by inhibiting actin polymerization (May et al., 1998). Before infection of *S*. Bareilly, Caco-2 cells were incubated at 37°C for 1 h with supplementation of 1 μg/ml cytochalasin D (Sigma). After 4 h incubation of *S*. Bareilly at 37°C, bacterial cells were added to the incubated Caco-2 cells at a MOI of 10 and additionally incubated for 30 min at 37°C for adhesion. After washing three times to remove non-attached bacteria, the cells were lysed using 1% Triton X-100 (Sigma), and a standard viable cell count with the serial dilution method was performed for CFU enumeration of attached *S.* Bareilly on the cells. This experiment was independently conducted in triplicate for statistical analysis.

### Biofilm formation

After *S*. Bareilly was cultivated in LB broth medium at 37°C for 12 h, 200 μl of the bacterial culture was placed into a 96-well cell culture plate (SPL Life Sciences, Korea) and incubated at 28°C for 5 days under static conditions. The biofilm-forming ability of the *S*. Bareilly isolates was assessed using a 96-well microtiter plate assay as previously described (Coffey and Anderson, 2014). The optical density of crystal violet-stained biofilms was measured at a wavelength of 570 nm. This experiment was independently conducted in triplicate for statistical analysis.

### Antimicrobial susceptibility test

Antimicrobial susceptibility was determined using the VITEK 2 system with a commercial AST-N169 card (bioMerieux Inc.) according to the manufacturer’s instructions; *Escherichia coli* ATCC 25922 was used as a control strain. Antibiotic susceptibility was interpreted according to the criteria issued by the Clinical and Laboratory Standards Institute (Clinical and Laboratory Standards Institute [CLSI], 2021). Seventeen antibiotics in the AST-N169 card were used for testing: ampicillin, amoxicillin/clavulanic acid, ampicillin/sulbactam, cefalothin, cefazolin, cefotetan, cefoxitin, cefotaxime, ceftriaxone, imipenem, amikacin, gentamycin, nalidixic acid, ciprofloxacin, tetracycline, chloramphenicol, and trimethoprim/sulfamethoxazole. The minimal inhibitory concentrations (MICs) of the representative *S*. Bareilly strains were determined using the broth microdilution method according to CLSI guidelines. Antibiotics were prepared with chloramphenicol (C; 2 to 64 μg/ml), ciprofloxacin (CIP; 0.25–8 μg/ml), erythromycin (ERY; 2–64 μg/ml), and tetracycline (TET; 1–32 μg/ml). The susceptibility of each isolate was determined using CLSI standards.

### Statistical analysis

Statistical analysis of all data was conducted to verify significance using Student’s *t*-test with a *P*-value < 0.05 by GraphPad Prism software v.5 (GraphPad Software Inc., USA).

## Results

### Isolation and serotyping of *Salmonella* food-borne disease outbreak strains

A total of 423 patients were diagnosed with *S. enterica* from 1,901 diarrheal patients, and 175 *Salmonella* strains were isolated from *S. enterica-*infected patients in Gyeonggi-do, South Korea from 2014 to 2018 (Supplementary Table 1). The isolates comprised 14 serotypes and were divided into the following somatic (O) antigen groups: 148 C type (13 C, 100 C1, 29 C2, and 6 C3), 24 B type, 2 D1 type, and 1 E4 type (Supplementary Table 2). The most predominantly isolated serotype was *S.* Livingstone (21.1%, *n* = 37), followed by *S.* Bareilly (17.7%, *n* = 31). To elucidate the origin and prevalence of *S.* Bareilly in South Korea, 31 *S.* Bareilly strains were isolated from 26 patients and five canteen employees in five different food-borne poisoning outbreaks in Gyeonggi-do, South Korea from 2014 to 2018, except 2017 (Table 1). There were no deaths in the hospital from these five *S.* Bareilly outbreaks, and symptoms were easily relieved after treatment with antibiotics (data not shown).

### Whole genome sequencing and phylogenetic analysis of *Salmonella* Bareilly

**FIGURE 1:**
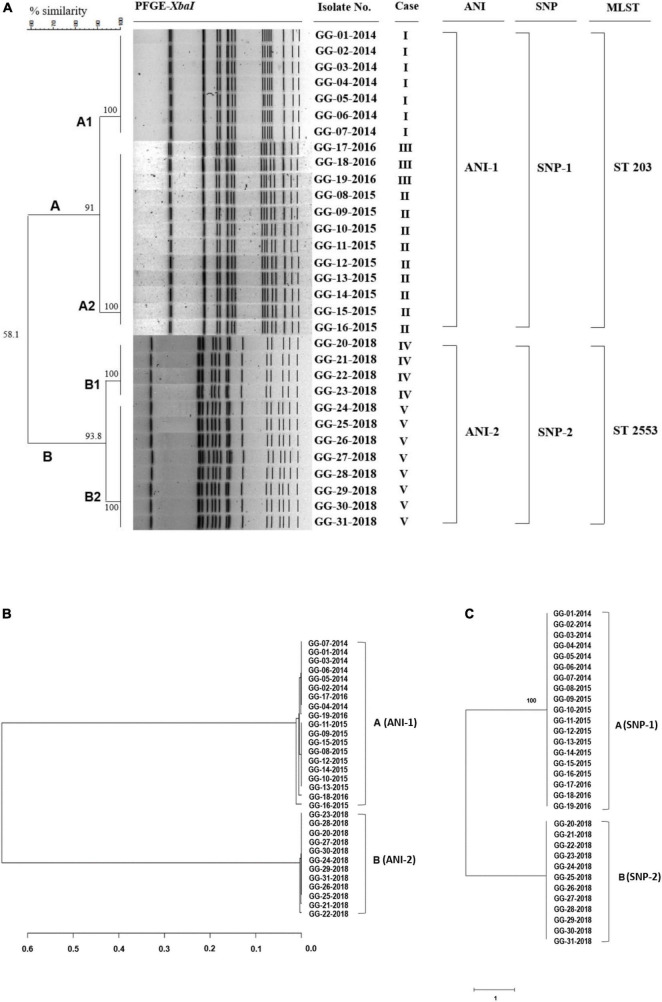
PFGE analysis, ANI phylogenetic tree, and SNP phylogenetic tree of 31 *S*. Bareilly clinical isolates from food-borne disease in South Korea. **(A)** Dendrogram of the PFGE-*Xba*I fragment pattern. Similarity (%) between patterns was calculated from the Dice index and is represented by the number beside the node. *In silico* multilocus sequence typing (MLST) analysis using seven gene sequences: *aroC, dnaN, hemD, hisD, purE, sucA*, and *thrA* showed two different groups of 31 *S.* Bareilly isolates, ST 203 and ST 2553. **(B)** The ANI phylogenetic tree of *S*. Bareilly. The evolutionary relatedness between *S*. Bareilly species were measured using the average nucleotide identity values. The average nucleotide identity (ANI) values were calculated using JSpecies by comparing whole genome sequences of *S*. Bareilly, which were fragmented into 1,020 bp, based on BLAST algorithm. The trees were constructed using R programming. **(C)** The SNP phylogenetic tree was analyzed using the neighbor joining method. Visualization of the tree was conducted using FigTree software (see Section “Materials and methods”).

To understand the phylogeny and genetic diversity of *S*. Bareilly outbreak strains, whole genome sequencing and the associated comparative phylogenetic tree analysis need to be performed. For this study, an *Xba*I digestion pattern-based PFGE method and WGS-based average nucleotide identity (ANI) analysis, single nucleotide polymorphism (SNP) analysis, and multilocus sequence typing (MLST) method were used, and the results were compared (Figure 1).

PFGE analysis showed that 31 *S.* Bareilly isolates were classified into two groups, A (*n* = 19) and B (*n* = 12), with 58.1% similarity. The A group strains were divided into A1 (7 strains) and A2 (12 strains) with 91% similarity, and the B group strains were divided into B1 (4 strains) and B2 (8 strains) with 93.8% similarity (Figure 1A). The A1 group strains were isolated from a food-borne outbreak in 2014, and the B1/B2 group strains were isolated from two outbreaks in 2018, suggesting that these outbreaks were caused by three different strains. In addition, while the A2 group strains originated from two outbreaks in 2015 and 2016, their PFGE patterns were identical, suggesting that the same pulsotype of *S.* Bareilly was the causative strain. Therefore, this PFGE analysis showed that four different outbreak groups were present in 31 *S.* Bareilly isolates.

The general genome characteristics of 31 *S*. Bareilly are summarized in Supplementary Table 3. The average genome assembly consisted of 4,682,537 bp with a G + C content of 52.2%, 4,363 coding sequences (CDSs), 7 rRNAs, and 73 tRNAs. Based on these WGSs of *S.* Bareilly strains, MLST analysis using seven house-keeping genes was performed showing two MLST sequence types, ST 203 and ST 2553. The ST 203 group strains containing 19 *S.* Bareilly strains were isolated from 2014 to 2016, indicating that the ST 203 group was identical to the A group. The ST 2553 group strains containing 12 *S.* Bareilly strains were isolated from 2018, indicating that it was identical to the B group. In addition, the ANI and SNP phylogenetic trees of all *S*. Bareilly strains were constructed using WGSs, showing two major groups in each phylogenetic tree (ANI-1 and ANI-2; SNP-1 and SNP-2) (Figure 1). The first major group (ANI-1 and SNP-1) contained 19 *S*. Bareilly strains isolated from food-borne outbreaks in 2014 to 2016, suggesting a shared origin. The second major group (ANI-2 and SNP-2) contained 12 *S.* Bareilly strains from the outbreak in 2018, suggesting a different origin from that of the 2014 to 2016 outbreaks. This result indicates that only two causative *S.* Bareilly strains are the origins of these outbreaks, unlike the previous PFGE analysis result. As the ANI and SNP analyses were performed on the genome level using WGSs of all *S.* Bareilly strains with high accuracy, there might be some interpretation errors with regard to the PFGE analysis results with low resolution.

Actually, the SNP analysis showed that there is no difference in SNPs between A and B groups (Figure 1C). However, the number of SNP counts was totally different even within the same group (Supplementary Table 6 and Supplementary Data Sheet 2). The range of SNP counts in A group is from 353 to 369 and that of SNP counts in B group is from 18750 to 18765, indicating that each strain has different SNP counts. Therefore, SNP counts are variable in all strains of A and B groups. In addition, the genome-wide SNP analysis with S. Bareilly FC745 as a reference strain showed that A and B groups are different in the SNP patterns (Supplementary Figure 2A). This SNP analysis revealed that A group-core SNPs are 122, B group-core SNPs are 18,510, and A/B group-core SNPs are 230 (Supplementary Figure 2B), indicating that there are group-specific SNP patterns between these two groups.

Furthermore, additional extended SNP analysis of the A/B group strains and various food-borne outbreak strains in other countries revealed that the A group strains and other strains from the UK, USA, Pakistan, and Sri Lanka belonged to the same SNP pattern group, suggesting that they share an origin strain (Supplementary Figure 1). However, the B group strains did not belong to any other SNP pattern group, suggesting that they are unique and present only in South Korea. Therefore, the A and B groups should be divided into two SNP pattern groups (Supplementary Figure 1). Based on these results, PFGE, MLST, ANI, and SNP analyses substantiate that there are two major groups, the A group and B group, in 31 *S.* Bareilly isolates in South Korea.

### Virulence factors of *Salmonella* Bareilly strains and their evolutionary relationship

**FIGURE 2:**
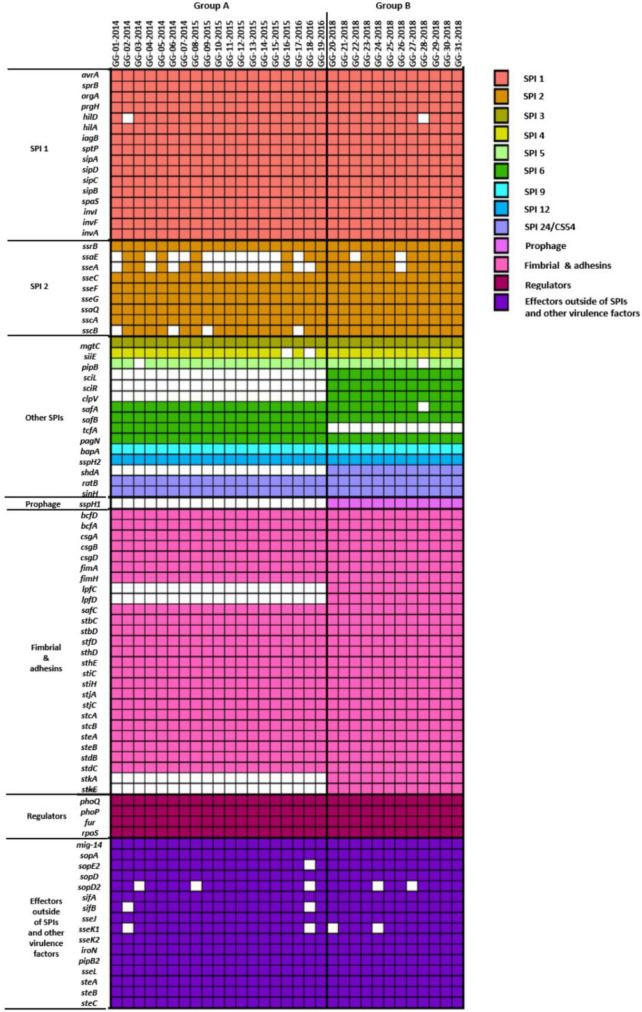
Heatmap of virulence genes present in 31 *S*. Bareilly isolates. Rows represent virulence genes and columns show each *S*. Bareilly isolate. The left side of the figure represents the following categories of virulence genes: SPIs, prophage, fimbria and adhesins, regulators, and effectors outside of SPIs and other virulence factors.

To understand the human infection of *S*. Bareilly, various *Salmonella* virulence factors including *Salmonella* pathogenicity islands (SPIs), prophage, fimbriae, regulators, and effectors were detected in the WGSs using the VFDB (Liu et al., 2019) and the presence of virulence factors in all *S.* Bareilly strains in the A and B groups was described in a heat map (Figure 2). Although the B group strains all possessed detected virulence factors, some genes were missing in the A group strains: *sci* gene cluster (cytoplasmic proteins), *clpV* (chaperone ATPase), *shdA* (AIDA autotransporter-like protein), *sspH1* (type III secretion system effector), *lpf* gene cluster (long polar fimbrial protein), and *stk* gene cluster (putative fimbrial protein) (Figure 2; Supplementary Table 4). However, *tcfA* (*S.* Typhi-specific colonizing factor) gene was missing in B group strains.

**FIGURE 3:**
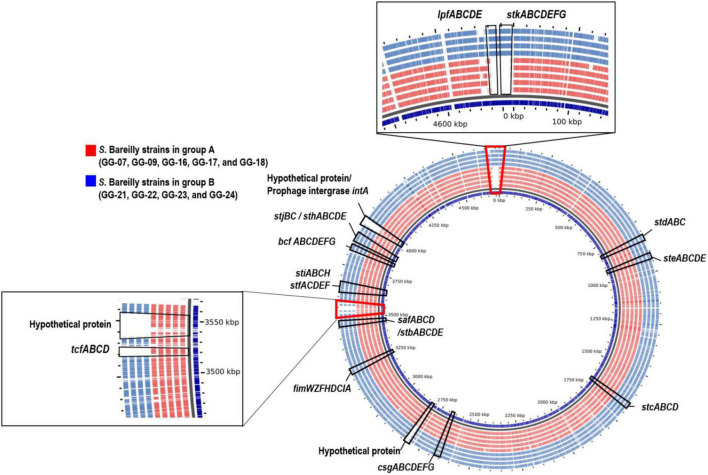
Pangenome analysis of representative *S*. Bareilly strains isolated in South Korea. *tcf* (Typhi colonizing factor), *lpf* (long polar fimbriae), *stk* (putative fimbrial protein), *csg* (thin aggregative fimbreiae), *fim* (Type 1 fimbriae), *bcf* (bovine colonization factor), *saf* (*Salmonella* atypical fimbriae), *stb, stc, std, ste, stf, sth, sti, stj* (putative chaperone-usher-dependent fimbrial operons).

To clarify the missing of these genes in *S.* Bareilly, comparative pangenome analysis was performed (Figure 3). For this analysis, nine identical genotype strains in five different outbreak case groups were selected (GG-07 in the case I group; GG-09, GG-16 in the case II group; GG-17, GG-18 in the case III group; GG-21, GG-22, GG-23 in the case IV group; GG-24 in the case V group; Figure 1; Supplementary Table 5). In general, fimbriae are involved in host cell attachment, colonization, and biofilm formation (Rehman et al., 2019). In both the A and B group strains, 12 essential gene clusters for fimbriae biosynthesis were detected in the genome: *fimAICDHFWYZ* (Type 1 fimbriae), *csgABCDEFG* (thin aggregative fimbriae), *safABCD* (*Salmonella* atypical fimbriae), *bcfABCDEFGH* (bovine colonization factor), *stbABCDE, stcABCD, stdABC, steABCDE, stfACDEF, sthABCDE, stiABCH*, and *stjBC* (putative chaperone-usher-dependent fimbrial operons), suggesting that *S.* Bareilly had the ability to form fimbriae (Figure 3). However, the *lpfCD* and *stkAE* genes encoding fimbrial proteins for fimbriae biosynthesis were found in the genome of *S.* Bareilly B group strains only (Figure 2). Subsequent pangenome analysis of these nine representative strains revealed that not only *lpfCD* and *stkAE* genes, but also whole gene clusters of *lpfABCDE* (long polar fimbriae) and *stkABCDEFG* (putative fimbrial protein) were missing in *S.* Bareilly A group strains (Figure 3). However, *tcfABCD* gene cluster is completely missing in B group strains. Based on these results, *stk* and *lpf* gene clusters are B group-specific, but *tcf* gene cluster is A group-specific, suggesting that their presence or absence could be an important criterion to determine the group-type (Figure 3).

**FIGURE 4:**
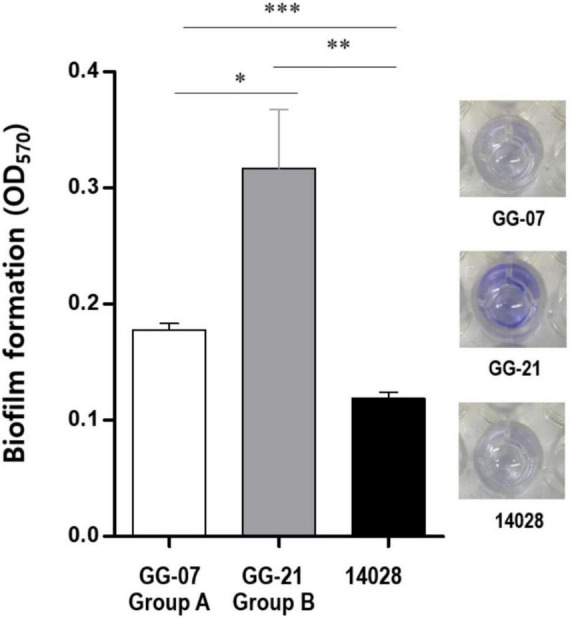
Biofilm formation of *S*. Bareilly. Biofilms attached to the plastic surface were stained with Crystal Violet after 5 days incubation at 28°C. All experiments were independently replicated in triplicate. NS, no significance; *p < 0.05; ^**^p < 0.005; ^***^p < 0.0005.

The loss of *lpf* and *stk* gene clusters in the *S.* Bareilly A group strains might have affected phenotypic characteristics of *S*. Bareilly strains. To elucidate this, each genotype strains in A and B group were selected (GG-07 in the A group; GG-21 in the B group) to observe the biofilm formation ability. Interestingly, the *S*. Bareilly GG-21 strain of the B group showed a significant higher biofilm formation at 28°C for 5 days than did *S*. Bareilly strain GG-07 of the A group and even *S.* Typhimurium ATCC 14028, probably due to reduction of biofilm formation by missing of *stk* gene cluster in the strains GG-07 (Figure 4). Although biofilm formation in the A group strains was weakened, *S.* Bareilly strains in both the A and B groups retained this ability, probably enabling survival under various environmental stresses and prevalence in a variety of food products (Abdallah et al., 2014).

To further understand the grouping of *S.* Bareilly and the evolutionary relationship of this serotype with other *Salmonella* serotypes, total 411 whole genome sequences of 80 different *Salmonella* serotypes (31 draft genome sequences of 31 *S.* Bareilly strains isolated from South Korea and 380 complete genome sequences of other serotype strains) were collected for comparative genomics and further pangenome analysis. For these comparative analyses, the presence of three different gene clusters (*stk, lpf*, and *tcf* gene clusters) in all collected genome sequences was confirmed and eight categories were grouped from C1 to C8, according to presence or absence of these three gene clusters (Supplementary Table 7). Interestingly, 12 B group strains of *S.* Bareilly were grouped to C3 with other serotypes of Cubana, Abony, Albany, Apapa, Stanleyville, Tennessee, Braenderup, and Thompson. This C3 has *stk* and *lpf* gene clusters but does not have *tcf* gene cluster. In addition, 19 A group strains of *S.* Bareilly were grouped to C6 with 20 other serotypes. This C6 has *tcf* gene cluster, but does not have *stk* and *lpf* gene clusters.

Based on these results, all serotype strains in C3 and C6 were collected and their ANI analysis was performed (Supplementary Figure 3). In the ANI tree, A and B group strains were in different branches, indicating that A and B groups are different. Interestingly, this ANI tree revealed that those B group strains were located in different branch from other serotypes in C3, suggesting that B group strains do not share the common ancestor with other serotypes in C3. In addition, although A group strains were in C6, they are located in the different branch comparing to other serotypes in C6. In addition, unlike other serotypes, *S.* Bareilly serotype strains from A/B groups and CFSAN were evolutionarily related in the tree, probably due to the same serotype. However, they were located in the different branches, suggesting that they may not share the ancestor of *S.* Bareilly. Interestingly, *S.* Bareilly FC745 used as a reference strain for SNP analysis belongs to C6 and is closely related to A group strains (Supplementary Figure 3). However, *S.* Bareilly RSE03 belongs to C1 without three gene clusters (*stk, lpf*, or *tcf* gene clusters), which is only one *S.* Bareilly strain in C1.

Previous SNP tree suggested that A group strains may be originated from other *S.* Bareilly outbreaks in UK, USA, India, Sri Lanka, Pakistan, and even Mexico (Supplementary Figure 1). However, while B group strains may be slightly related with CFSAN strains, they are located in the separate branch and different from A group and CFSAN strains in *S.* Bareilly serotype (Supplementary Figure 3). The previous SNP tree also suggested that B group strains may be a new genotype, supporting this. Based on these results, although B group strains belong to C3, they are very different from other serotype strains of C3 in the tree. And B group strains are not related to other *S.* Bareilly serotype strains. Therefore, these all results suggest that B group strains may be a new genotype of *S.* Bareilly.

### Antimicrobial susceptibility of *Salmonella* Bareilly isolates

To investigate the antibiotic susceptibilities of 31 isolated *S.* Bareilly strains in South Korea, VITEK 2 analysis was performed, showing that all strains were susceptible to 17 antibiotics on the AST-N169 card (Supplementary Table 8). However, five antibiotic resistance genes such as *golS* (copper efflux regulator for chloramphenicol), *mdsA* (multi-drug and metal efflux complex for chloramphenicol), *mdtK* (multidrug and toxic compound extrusion transporter for ciprofloxacin), *crp* (cAMP receptor protein for erythromycin), and *sdiA* (cell division regulatory protein for tetracycline) were detected in their genome using the Comprehensive Antibiotic Resistance Database (CARD) (Alcock et al., 2020). To confirm the real antibiotic resistance activity of these four antibiotics, the minimal inhibitory concentrations (MICs) were determined (Supplementary Table 9). Interestingly, the MICs of chloramphenicol, ciprofloxacin, and tetracycline were 4, ≤ 0.25, and ≤1 μg/ml, respectively, which were lower than the break points of CLSI (chloramphenicol, ≥32 μg/ml; ciprofloxacin, ≥4 μg/ml; tetracycline ≥16 μg/ml), suggesting that all *S.* Bareilly strains in South Korea are susceptible to these three antibiotics. However, the MICs of erythromycin in all tested strains were >64 μg/ml. Due to no break point of CLSI to erythromycin in *Salmonella*, this did not clearly confirm if all tested strains were susceptible to erythromycin (Hassing et al., 2014).

## Discussion

Although *S. enterica* serovars including *S.* Typhimurium, *S.* Enteritidis, *S.* Typhi, and *S.* Paratyphi have been well-known as major food-borne pathogens for salmonellosis, *S.* Bareilly was first identified as a pathogenic serovar causing food-borne outbreaks since 2010 in UK (Cleary et al., 2010; Hoffmann et al., 2016). Since then, this serovar has been emerged as a major food-borne pathogen for various food-borne outbreaks in Western countries and has become one of the most frequent serovars of enteric salmonellosis in the United States (Centers for Disease Control and Prevention [CDC], 2018; Dewey-Mattia et al., 2018). However, in Eastern countries, *S.* Bareilly was first detected in South Korean poultry farms in 2013–2014 (Im et al., 2015; Kim et al., 2015). While *S.* Bareilly infection has widely spread in the last decade, the pathogenic and taxonomical characterization and even its prevalence have not been investigated. Therefore, it is necessary to study the genome sequence-based taxonomical relativeness, molecular pathogenesis, and antibiotic resistance analysis of this serovar of *Salmonella.*

A statistical report regarding *Salmonella* outbreaks in the United States showed that most *Salmonella* outbreaks occurred from late spring to early fall (Akil et al., 2014). And, most of the *Salmonella* poisoning cases occurred between March and October in South Korea, supporting this (Supplementary Table 1). Interestingly, 31 *S.* Bareilly strains in this study were isolated from five food-borne outbreaks only from August to October in South Korea between 2014 and 2018 (Table 1). However, it is not clearly understood yet why this serovar propagates between late summer and early fall, comparing to other serovars of *Salmonella*. Previously, *S.* Bareilly was detected in meat products (tuna, chicken) and fresh vegetables (bean sprout). In South Korea, *S*. Bareilly strains in ST 203 were observed in chicken meat (Choi et al., 2015; Park et al., 2017). In this study, the A group including outbreak cases I and II might be associated with meat (e.g., beef and pork bulgogi, pork cutlet, and stir-fried pork), and the B group including outbreak cases IV and V might be associated with kimbap containing stir-fried egg produced by the same manufacturing facility (Figure 1 and Table 1). Therefore, the original foods for the *S.* Bareilly outbreaks were not clearly determined in this study. This result suggests that the food sources of *S.* Bareilly infection could vary and were not limited to meat or fresh vegetables.

In this study, between 2014 and 2018 in Gyeonggi-do, South Korea, 175 *Salmonella enterica* strains were isolated and identified from 423 *Salmonella-*positive patients (Supplementary Table 1). Among them, *S.* Livingsteone was isolated with the highest number from three different food-borne outbreaks in 2014. *S.* Livingstone was first isolated from patients staying at a hotel in Victoria Falls, Zambia in 1951 (Picton et al., 1953) and it was also isolated from animals and feedstuffs (Eld et al., 1991). Since then, *S.* Livingstone was determined as the causative agent of large-scale human salmonellosis outbreaks occurred in Europe (Old et al., 1994; Guerin et al., 2004; Eriksson et al., 2005). Interestingly, previous 2014 prevalence study for *Salmonella* in South Korea reported that *S.* Livingstone was the third most common serotype (12.8%) (Korea Disease Control and Prevention Agency [KDCA], 2015). However, this serotype was detected in South Korea with a few regional and periodic limitations: (1) only Gyeonggi-do and Seoul (2) year 2014 (3) only domestic outbreaks, not from international travelers. Based on this, *S.* Livingstone might be detected and isolated from specific food-borne outbreaks, not prevalent ones. In this study, *S.* Livingstone was also detected and isolated as the most common *Salmonella* serotype from only Suwon and Icheon region of Gyeonggi-do in 2014, supporting this (Supplementary Table 1). However, in this study, *S.* Bareilly was detected and isolated with the second highest number from broader regions and year 2014–2018, unlike *S.* Livingstone. Therefore, *S.* Bareilly was selected for further investigation, because it has been more prevalent and widely spread in South Korea during last decade.

This surveillance study revealed that *S.* Bareilly consists of two distinct groups (A and B groups) in South Korea, confirmed by ANI, SNP, and MLST analyses with WGS information (Figure 1). Interestingly, the A group strains shared similar SNP pattern and were located in the same branch of the SNP tree as the UK, India, Mexico, Pakistan, Sri Lanka, and the USA strains, suggesting that they share a common ancestor with the UK and other countries. Conversely, B group strains were located in a separate branch of the SNP tree, indicating that this group is present only in South Korea (Supplementary Figure 1). Further comparative analysis of the A and B group strains showed that the A group strains lost some important genes associated with invasion, colonization, survival, and even biofilm formation, possibly affecting their pathogenesis. In addition, after the A group strains were identified in food-borne outbreaks between 2014 and 2016, B group strains were detected in *S.* Bareilly food-borne outbreaks in 2018. Based on this, a new type of *S.* Bareilly in the B group might have exhibited greater pathogenetic activities regarding human infection and survival. Therefore, further pathogenesis studies with distinct *S.* Bareilly B group strains needs to be elucidated to control this new pathogen type.

Whole genome sequences of *S.* Bareilly were analyzed to detect their virulence factors in genome level. Among the SPIs, *S.* Bareilly strains had nine SPIs containing SPI-1, SPI-2, and other SPIs (SPI-3, SPI-4, SPI-5, SPI-6, SPI-9, SPI-12, and SPI-24). SPI-1 and SPI-2 encoded the Type 3 secretion system (T3SS) associated with host cell invasion and intracellular survival, key functions for human infection (Cheng et al., 2019). Other SPIs (SPI-3, SPI-4, SPI-5, and SPI-6) can be involved in host invasion and intracellular survival, but the others (SPI-9, SPI-12, and SPI-24) are involved in cell adherence and colonization (Cheng et al., 2019). Based on the functions of SPIs, *S.* Bareilly had all required functions for human infection, comprising host cell adherence, colonization, invasion, and intracellular survival. In addition, most of the core fimbriae genes were present in *S*. Bareilly strains (Figure 2).

The *S.* Bareilly genomes also possessed several global transcriptional regulators associated with stress response and survival (Figure 2). The *phoPQ* two-component system acts as a sensor kinase (PhoQ) and a transcription regulator (PhoP) for environmental response, especially those associated with control of several gene expressions regarding bacterial survival from phagocytosis (Dalebroux and Miller, 2014). In addition, *fur, rpoE*, and *rpoS* genes were suggested to be related to global regulation of cationic metabolism and stress response (Ibanez-Ruiz et al., 2000; Miticka et al., 2003; Troxell and Hassan, 2013). Therefore, these regulators might be involved in the regulation of specific genes regarding the pathogenicity of *S.* Bareilly. In addition, various effector proteins generally secreted *via* T3SS were encoded in the genomes of *S.* Bareilly, probably regarding virulence activity (Figure 2). The *sopADD2E2* and *sifAB* genes located outside SPI-1/SPI-2 were suggested to be associated with bacterial survival in host tissues and bacterial invasion in host cells, respectively (Valenzuela et al., 2021). The *sseJK1K2L* genes located outside SPI-2 were previously suggested to be involved in *Salmonella-*containing vacuole (SCV) biogenesis for phagosomes to evade host phagocytosis (Ohlson et al., 2005). Therefore, these genes might be key components for bacterial survival and replication in host cells. The *steABC* genes were also suggested to be related to control of SCV membrane dynamics for immune response suppression of host cells to help evade host phagocytosis (Domingues et al., 2014). Therefore, these effectors might have played important roles in *Salmonella* virulence for promotion of host cell invasion, survival, and replication of *S.* Bareilly. The *sci* gene cluster in the Type 6 secretion system (T6SS) is associated with membrane localization for extracellular processes such as secretion and organelle biosynthesis (Folkesson et al., 2002). In addition, deletion of this gene cluster affected invasion and survivability in epithelial cells. Therefore, this *sci* gene cluster might be a core gene of T6SS for intracellular toxin secretion after cell invasion. In addition, the *clpV* gene encoding chaperone ATPase is frequently detected with the *sci* gene cluster in the T6SS. This gene was previously suggested to support hemolysin-coregulated protein (Hcp) and T6SS spike protein (VgrG2) for toxin secretion *via* T6SS (Bönemann et al., 2009). To elucidate the role of *clpV* gene, it was deleted and showed the lowering of bacterial colonization on the surface of epithelial cells (Pezoa et al., 2013). Therefore, the *sci* gene cluster and *clpV* gene in T6SS might have been involved in colonization and invasion of epithelial cells to support intracellular toxin secretion *via* T6SS. Subsequent cell adhesion and invasion activity tests on Caco-2 human epithelial cells with the GG-07 strain of the A group and the GG-21 strain of the B group were performed to support this. Interestingly, the cell adhesion and invasion activities of GG-07 were lower than those of GG-21 (Supplementary Figures 4A,B). Absence of *sci* gene cluster in A group strain support these low cell adhesion and invasion activities comparing to B group strains (Figure 2). The *lpf* gene cluster encoding long polar fimbriae plays a role in adhesion of *Salmonella* to murine Peyer’s patches (Bäumler et al., 1996a) and biofilm formation (Bäumler et al., 1996b; Weening et al., 2005; Ledeboer et al., 2006). The mutation of this gene cluster exhibited complete loss of the ability to form biofilms on chicken intestinal tissue or an intermediate loss of the ability to form biofilms on tissue culture cells and plastic surfaces (Ledeboer et al., 2006). In addition, the function of *stk* gene cluster is not clearly understood, but it was suggested to be associated with fimbriae biosynthesis regarding cell adhesion and biofilm formation. It was recently reported that heterologous expression of *S.* Paratyphi A *stkF* gene in *E. coli* enhanced host cell adhesion for further host invasion, suggesting that the missing of *stk* gene cluster may be involved in loss or weakness of host cell adhesion and biofilm formation (Tawfick et al., 2020). Therefore, loss of these gene clusters in the *S.* Bareilly A group strains might have affected host cell adhesion and biofilm formation. Interestingly, a comparative host cell adhesion and biofilm formation assay showed that B group strain GG-21 had higher activities than A group strain GG-07, probably due to reduction of cell adhesion and biofilm formation by missing of *stk* gene cluster in the strains GG-07 (Supplementary Figure 4A; Figure 4). In addition, while *sci* gene cluster and *clpV* gene are present in B group strain GG-21, they are missing in A group strain GG-07. Because invasion activity may be associated with *sci* gene cluster and *clpV* gene as previously discussed, the invasion activity of A group strain GG-07 was much lower than those of GG-21 (Supplementary Figure 4B). Therefore, the emergence of B group *S*. Bareilly strains has the potential for increased risk with regard to disease outbreaks through its virulence-associated characteristics. The *shdA* gene was detected in the SPI24 of the B group strains of *S.* Bareilly (Figure 2). This gene encoded an AIDA (adhesin in diffuse adherence) autotransporter-like protein probably for cell adhesion (Kingsley et al., 2003). Previously, this gene was found on the SPI24/CS54 island of *S.* Typhimurium and the *shdA* deletion mutant showed reduced colonization of the cecum and Peyer’s patches (Kingsley et al., 2003). Missing of this gene in the A group strains affected colonization of the epithelial cells, similar to the *clpV* gene. Therefore, a reduction of adhesion activity of the A group strain GG-07 without the *clpV* and *shdA* genes supported this (Supplementary Figure 4A). In addition, while the *sspH2* gene was detected in all *S.* Bareilly genomes, the *sspH1* gene was detected in the prophage region of B group strains only (Figure 2). In general, *SspH1* had a restricted distribution in *Salmonella* serotypes, while *SspH2* was widely distributed (Quezada et al., 2009). Each of SPI1 and SPI2 had a T3SS; each T3SS had two *sspH1/sspH2* genes encoding *Salmonella* effectors, probably targeting T3SS (Miao et al., 1999). While their exact function is unknown, these genes encoding E3 ubiquitin-protein ligase domains were suggested to be associated with alteration of host cell physiology and promotion of bacterial survival in host tissues *via* interference of the host’s ubiquitination pathway (Quezada et al., 2009). Genome sequence analysis of *S.* Bareilly isolates revealed the presence of the *sspH1* gene in the prophage region of B group strains only, indicating that this gene can be transferrable to other *S.* Bareilly strains for virulence. The *tcfABCD* gene cluster encoding *S.* Typhi-specific colonizing factor was missing in the B group strains (Figure 3). While this gene cluster was previously only detected in *S*. Typhi (Folkesson et al., 1999), subsequent *Salmonella* genome studies revealed that it was also detected in some other non-typhoidal *Salmonella* serotypes such as Choleraesuis, Schwarzengrund, Heidelberg, Virchow, Montevideo (Townsend et al., 2001; Bronowski and Winstanley, 2009; den Bakker et al., 2011), and some Bareilly strains (Kim and Lee, 2017), suggesting possible gene transfer from *S.* Typhi. However, previous *tcf-*knockout mutations revealed no significant difference in adhesion and invasion levels between the mutant and wild-type strains (Leclerc et al., 2016). Therefore, the *tcf* gene cluster might not play an important role in the pathogenesis of *S.* Bareilly.

As previously reported, *S.* Bareilly isolates in South Korea are susceptible to various antibiotics (Im et al., 2015). In this study, all isolates of *S.* Bareilly are highly susceptible to antibiotics (Supplementary Table 8). However, other studies regarding antibiotic resistance in *Salmonella* during similar periods to those in this study showed that other serovars of *Salmonella* had high resistance to various antibiotics. The first surveillance study of *S.* Virchow from South Korean patients between 2005 and 2014 revealed that cefotaxime-resistant *S.* Virchow had rapidly increased since its first detection in 2011 (Kim et al., 2016). These CTX-M-15 type strains also showed various additional resistance against ampicillin, cephalothin, gentamycin, nalidixic acid, and tetracycline, indicating multi-drug resistance (MDR). In addition, a similar surveillance study of *S.* Virchow from food-producing animals in South Korea from 2010 to 2017 showed rapid emergence of extended-spectrum cephalosporin (ESC)-resistant strains (63.8% of all *S.* Virchow isolates), mostly from chicken samples between 2013 and 2015 (Na et al., 2020). Interestingly, all the ESC-resistant strains exhibited resistance activity of CTX-M-15 type (87.0%) and CMY-2 type (13.0%). In addition, these ESC-resistant strains showed other antibiotic resistance activity such as streptomycin, ampicillin, nalidixic acid, and tetracycline, supporting the MDR of *S.* Virchow. Furthermore, the most recent surveillance study revealed that *Salmonella enterica* isolates in South Korea between 2016 and 2017 were highly resistant to ampicillin, tetracycline, chloramphenicol, gentamicin, trimethoprim/sulfamethoxazole, cefotaxime, and ceftazidime (Kim et al., 2022). In particular, the most common MDR serotype was *S.* I 4,[5],12:i:-, followed by *S*. Typhimurium and *S*. Albany. This study also showed that the spread of *S*. Bareilly has recently emerged in South Korea, but the isolates exhibited low antibiotic resistance. As discussed above, while other *Salmonella* serotype strains had MDR activities in the same period in South Korea, *S.* Bareilly exhibited low antibiotic resistance, suggesting that human infection with this pathogenic bacterium could be easily treated with antibiotics. The *S.* Bareilly-infected patients in this study rapidly recovered after antibiotic treatment without side effects (data not shown). Therefore, it might be necessary to elucidate why *S.* Bareilly is susceptible to various antibiotics unlike other *Salmonella* serotypes.

Consequently, this study provides extensive genetic and evolutionary insights into the pathogenesis of a new serovar bacterium of *Salmonella* for further food safety research.

## Data availability statement

The data presented in the study are deposited in the NCBI Sequence Read Archive (SRA) under BioProject accession number PRJNA614548.

## Author contributions

J-HL and SR: conceptualization, writing–review and editing, supervision, project administration, and funding acquisition. NP, J-GK, HN, and SL: investigation and statistical analysis. NP, J-GK, and J-HL: validation and formal analysis. NP and J-GK: visualization. NP and J-HL: writing–original draft. All authors contributed to the article and approved the submitted version.

## Footnotes

1
https://server.gview.ca/
