# Supplementary material for: Discovery and characterization of a new genotype of Salmonella enterica serovar Bareilly isolated from diarrhea patients of food-borne outbreaks
Source: Front Microbiol. 2022 Oct 26;13:1024189. doi: 10.3389/fmicb.2022.1024189 (PMC9643863; doi:10.3389/fmicb.2022.1024189)

**Supplementary Information**

**Discovery and Characterization of a** **New Genotype of *Salmonella enterica* serovar Bareilly Isolated from Diarrhea Patients of Food-borne Outbreaks**

**Nanjoo Park^1,2,3,4,5†^, Joon-Gi Kwon^1,2,3,4†^, Hongjun Na^1,2,3,4,6^, Sohyun Lee^5^,**

**Ju-Hoon Lee^1,2,3,4*^, and Sangryeol Ryu^1,2,3,4*^**

^1^Department of Food and Animal Biotechnology, Seoul National University, Seoul, South Korea

^2^Department of Agricultural Biotechnology, Seoul National University, Seoul, South Korea

^3^Research Institute of Agriculture and Life Sciences, Seoul National University, Seoul, South Korea

^4^Center for Food and Bioconvergence, Seoul National University, Seoul, South Korea

^5^Gyeonggi-do Research Institute of Health & Environment, Suwon, South Korea

^6^Research and Development Center, Sanigen Co. Ltd., Anyang, South Korea

**^†^ These authors have contributed equally to this work and share first authorship**

**^*^ Correspondence:**

Dr. Ju-Hoon Lee / [juhlee@snu.ac.kr](mailto:juhlee@snu.ac.kr)

Dr. Sangryeol Ryu / [sangryu@snu.ac.kr](mailto:sangryu@snu.ac.kr)

**TABLE S1.** Summary of *Salmonella enterica* outbreaks in Gyeonggi-do, South Korea from 2014 to 2018.

| **Time** | **City** | **No. of patients** | **No. of isolates** | ***S. enterica* serovar** |
| --- | --- | --- | --- | --- |
| Mar-2014 | Suwon | 20 | 1 | Livingstone (1) |
| Mar-2014 | Suwon | 38 | 22 | Livingstone (21), Montevideo (1) |
| Mar-2014 | Icheon | 32 | 16 | Livingstone (15), Montevideo (1) |
| July-2014 | Anyang | 16 | 9 | Thompson (9) |
| July-2014 | Uiwang | 3 | 1 | Thompson (1) |
| Sep-2014 | Uiwang | 2 | 1 | Braenderup (1) |
| Sep-2014 | Yongin | 8 | 4 | Braenderup (4) |
| **Sep-2014** | Ansan | 13 | 7 | **Bareilly** (7) |
| **Aug-2015** | Hwaseong | 17 | 9 | **Bareilly** (9) |
| Aug-2015 | Yongin | 3 | 2 | Enteritidis (2) |
| June-2016 | Gwangmyeong | 37 | 26 | Bovismorbificans (26) |
| Aug-2016 | Bucheon | 14 | 2 | Typhimurium (2) |
| Aug-2016 | Gunpo | 1 | 1 | Infantis (1) |
| Aug-2016 | Yeoju | 10 | 3 | Blockley (3) |
| Aug-2016 | Seongnam | 2 | 2 | Infantis (2) |
| Sep-2016 | Yongin | 6 | 1 | I 4,[5],12:i:- (1) |
| **Oct-2016** | Yongin | 37 | 3 | **Bareilly** (3) |
| Apr-2017 | Suwon | 8 | 1 | Dessau (1) |
| May-2017 | Gimpo | 6 | 6 | Langensalza (6) |
| Nov-2017 | Incheon | 12 | 9 | Infantis (9) |
| Apr-2018 | Hwaseong | 28 | 16 | I 4,[5],12:i:- (16) |
| Sep-2018 | Pyeongtaek | 7 | 2 | Motevideo (2) |
| Sep-2018 | Yongin | 24 | 13 | Thompson (13) |
| Sep-2018 | Bucheon | 11 | 5 | Typhimurium (5) |
| **Oct-2018** | Gunpo | 27 | 8 | **Bareilly** (8) |
| **Oct-2018** | Gimpo | 12 | 4 | **Bareilly** (4) |
| Oct-2018 | Suwon | 29 | 1 | Othmarschen (1) |
| Total | | 423 | 175 |  |

**TABLE S2.** Distribution of *Salmonella enterica* serovars isolated from foodborne disease patients from 2014 to 2018, in South Korea.

| Serovar | Somatic antigen | Year | Total number (%) |
| --- | --- | --- | --- |
| Livingstone | C1 | 2014 | 37 (21.1) |
| Bareilly | C1 | 2014, 2015, 2016, 2018 | 31 (17.7) |
| Bovismorbificans | C2 | 2016 | 26 (14.9) |
| Thompson | C1 | 2014, 2018 | 23 (13.1) |
| 4,[5],12:i:- | B | 2016, 2017 | 17 (9.7) |
| Infantis | C | 2016, 2017 | 12 (6.9) |
| Typhimurium | B | 2016, 2018 | 7 (4.0) |
| Langensalza | C3 | 2017 | 6 (3.4) |
| Braenderup | C1 | 2014 | 5 (2.9) |
| Montevideo | C1 | 2014, 2018 | 4 (2.3) |
| Blockley | C2 | 2016 | 3 (1.7) |
| Enteritidis | D1 | 2015 | 2 (1.1) |
| Dessau | E4 | 2017 | 1 (0.6) |
| Othmarschen | C | 2018 | 1 (0.6) |
| Total | | | 175 (100) |

**TABLE S3.** Whole-genome sequencing information of *S*. Bareilly strains in this study.

| Strain | Genome size (bp) | GC contents (%) | Number of contig | Number of CDS | Number of rRNA | Number of tRNA |
| --- | --- | --- | --- | --- | --- | --- |
| GG01 | 4,693,527 | 52.22 | 21 | 4,378 | 7 | 74 |
| GG02 | 4,687,458 | 52.25 | 50 | 4,372 | 7 | 72 |
| GG03 | 4,692,401 | 52.23 | 35 | 4,377 | 7 | 74 |
| GG04 | 4,694,046 | 52.22 | 19 | 4,377 | 7 | 74 |
| GG05 | 4,693,777 | 52.22 | 26 | 4,380 | 7 | 75 |
| GG06 | 4,693,697 | 52.22 | 21 | 4,378 | 7 | 74 |
| GG07 | 4,694,471 | 52.22 | 22 | 4,381 | 7 | 75 |
| GG08 | 4,624,875 | 52.28 | 33 | 4,276 | 7 | 72 |
| GG09 | 4,624,467 | 52.27 | 20 | 4,283 | 7 | 70 |
| GG10 | 4,625,617 | 52.27 | 18 | 4,283 | 7 | 72 |
| GG11 | 4,625,947 | 52.27 | 20 | 4,284 | 7 | 72 |
| GG12 | 4,626,583 | 52.27 | 20 | 4,285 | 7 | 72 |
| GG13 | 4,626,757 | 52.27 | 20 | 4,285 | 7 | 72 |
| GG14 | 4,627,251 | 52.27 | 18 | 4,282 | 7 | 75 |
| GG15 | 4,626,838 | 52.27 | 19 | 4,280 | 7 | 72 |
| GG16 | 4,631,890 | 52.27 | 28 | 4,285 | 10 | 74 |
| GG17 | 4,674,044 | 52.23 | 17 | 4,352 | 7 | 74 |
| GG18 | 4,649,749 | 52.31 | 43 | 4,337 | 7 | 72 |
| GG19 | 4,673,452 | 52.23 | 27 | 4,355 | 7 | 74 |
| GG20 | 4,699,387 | 52.23 | 23 | 4,375 | 8 | 77 |
| GG21 | 4,693,356 | 51.21 | 26 | 4,371 | 3 | 66 |
| GG22 | 4,703,177 | 52.21 | 21 | 4,383 | 8 | 75 |
| GG23 | 4,699,268 | 52.22 | 23 | 4,382 | 7 | 72 |
| GG24 | 4,731,184 | 52.24 | 58 | 4,439 | 6 | 73 |
| GG25 | 4,733,723 | 52.19 | 27 | 4,438 | 3 | 66 |
| GG26 | 4,738,774 | 52.19 | 20 | 4,448 | 7 | 73 |
| GG27 | 4,739,115 | 52.2 | 27 | 4,445 | 7 | 73 |
| GG28 | 4,729,865 | 52.23 | 47 | 4,426 | 8 | 73 |
| GG29 | 4,734,677 | 52.22 | 27 | 4,435 | 7 | 73 |
| GG30 | 4,730,217 | 52.2 | 22 | 4,445 | 7 | 73 |
| GG31 | 4,739,045 | 52.19 | 23 | 4,445 | 7 | 73 |

**TABLE S4.** Group-specific virulence factors of *S*. Bareilly strains in group B.

| **Gene** | **Description** | **Percent Identity (%)** | **Query Cover**  **(%)** |
| --- | --- | --- | --- |
| ***clpV*** | chaperone ATPase | 99.28 | 100 |
| ***lpfA*** | long polar fimbria protein LpfA | 100 | 100 |
| ***lpfB*** | long polar fimbrial chaperone protein LpfB | 99.714 | 100 |
| ***lpfC*** | long polar fimbrial outer membrane usher protein | 99.763 | 100 |
| ***lpfD*** | long polar fimbrial protein LpfD | 99.815 | 100 |
| ***lpfE*** | long polar fimbrial minor subunit LpfE, adhesin | 100 | 100 |
| ***sciA*** | cytoplasmic protein | 99.337 | 100 |
| ***sciB*** | cytoplasmic protein | 99.699 | 100 |
| ***sciC*** | cytoplasmic protein | 99.363 | 100 |
| ***sciD*** | cytoplasmic protein | 98.99 | 100 |
| ***sciE*** | cytoplasmic protein | 98.667 | 100 |
| ***sciF*** | cytoplasmic protein | 99.336 | 100 |
| ***sciH*** | cytoplasmic protein | 99.448 | 100 |
| ***sciI*** | cytoplasmic protein | 99.139 | 100 |
| ***sciJ*** | cytoplasmic protein | 99.329 | 100 |
| ***sciK*** | cytoplasmic protein | 99.177 | 100 |
| ***sciL*** | cytoplasmic protein | 99.794 | 100 |
| ***sciM*** | cytoplasmic protein | 100 | 100 |
| ***sciN*** | outer membrane lipoprotein | 99.441 | 100 |
| ***sciO*** | cytoplasmic protein | 99.405 | 100 |
| ***sciP*** | hypothetical protein | 99.54 | 100 |
| ***sciQ*** | inner membrane protein | 98.579 | 100 |
| ***sciR*** | Shiga-like toxin A subunit | 99.769 | 100 |
| ***sciS/icmF-like*** | inner membrane protein | 99.147 | 100 |
| ***sciT*** | cytoplasmic protein | 98.956 | 97.085 |
| ***sciU*** | cytoplasmic protein | 98.801 | 100 |
| ***sciV*** | cytoplasmic protein | 99.617 | 100 |
| ***shdA*** | AIDA autotransporter-like protein | 97.194 | 100 |
| ***sspH1*** | type III secretion system effector SspH1,  E3 ubiquitin ligase | 97.917 | 100 |
| ***stkA*** | major fimbrial subunit | 99.835 | 100 |
| ***stkB*** | putative chaperone protein EcpD | 99.469 | 100 |
| ***stkC*** | putative outer membrane usher protein | 99.346 | 100 |
| ***stkD*** | putative fimbrial protein | 98.674 | 100 |
| ***stkE*** | putative fimbrial protein | 99.642 | 100 |
| ***stkF*** | putative fimbrial protein | 99.465 | 100 |
| ***stkG*** | putative fimbrial protein | 99.812 | 100 |

**TABLE S5.** Whole-genome ANI values of S. Bareilly strains for each foodborne disease outbreak cases.

| **Outbreak**  **case** | **Strain** | **GG**  **01** | **GG**  **02** | **GG**  **03** | **GG**  **04** | **GG**  **05** | **GG**  **06** | **GG**  **07*** | **GG**  **08** | **GG**  **09*** | **GG**  **10** | **GG**  **11** | **GG**  **12** | **GG**  **13** | **GG**  **14** | **GG**  **15** | **GG**  **16*** | **GG**  **17*** | **GG**  **18*** | **GG**  **19** | **GG**  **20** | **GG**  **21*** | **GG**  **22*** | **GG**  **23*** | **GG**  **24*** | **GG**  **25** | **GG**  **26** | **GG**  **27** | **GG**  **28** | **GG**  **29** | **GG**  **30** | **GG**  **31** |
| --- | --- | --- | --- | --- | --- | --- | --- | --- | --- | --- | --- | --- | --- | --- | --- | --- | --- | --- | --- | --- | --- | --- | --- | --- | --- | --- | --- | --- | --- | --- | --- | --- |
| **Case I** | **GG01** | --- | 100 | 100 | 100 | 100 | 100 | 100 | 100 | 100 | 100 | 100 | 100 | 100 | 99.99 | 100 | 99.98 | 100 | 99.99 | 100 | 99.35 | 99.35 | 99.34 | 99.35 | 99.34 | 99.34 | 99.34 | 99.36 | 99.33 | 99.34 | 99.34 | 99.34 |
|  | **GG02** | 100 | --- | 100 | 100 | 100 | 100 | 100 | 99.99 | 99.99 | 99.99 | 99.99 | 99.99 | 99.99 | 99.99 | 99.99 | 99.98 | 100 | 99.99 | 99.99 | 99.34 | 99.35 | 99.34 | 99.35 | 99.34 | 99.34 | 99.33 | 99.36 | 99.33 | 99.34 | 99.34 | 99.35 |
|  | **GG03** | 100 | 100 | --- | 100 | 100 | 100 | 100 | 99.99 | 99.99 | 99.99 | 99.99 | 99.99 | 99.99 | 99.99 | 99.99 | 99.98 | 100 | 99.99 | 100 | 99.34 | 99.34 | 99.34 | 99.34 | 99.34 | 99.33 | 99.33 | 99.35 | 99.33 | 99.34 | 99.33 | 99.34 |
|  | **GG04** | 100 | 100 | 100 | --- | 100 | 100 | 100 | 99.99 | 99.99 | 99.99 | 99.99 | 99.99 | 99.99 | 99.99 | 99.99 | 99.98 | 100 | 99.99 | 99.99 | 99.33 | 99.34 | 99.33 | 99.34 | 99.33 | 99.33 | 99.32 | 99.35 | 99.32 | 99.33 | 99.33 | 99.33 |
|  | **GG05** | 100 | 100 | 100 | 100 | --- | 100 | 100 | 99.99 | 99.99 | 99.99 | 99.99 | 99.99 | 99.99 | 99.99 | 99.99 | 99.99 | 100 | 99.99 | 100 | 99.34 | 99.35 | 99.34 | 99.35 | 99.34 | 99.34 | 99.33 | 99.36 | 99.33 | 99.34 | 99.34 | 99.34 |
|  | **GG06** | 100 | 100 | 100 | 100 | 100 | --- | 100 | 99.99 | 99.99 | 99.99 | 99.99 | 99.99 | 99.99 | 99.99 | 99.99 | 99.99 | 100 | 99.99 | 100 | 99.34 | 99.34 | 99.33 | 99.34 | 99.33 | 99.33 | 99.33 | 99.35 | 99.33 | 99.34 | 99.33 | 99.34 |
|  | **GG07*** | 100 | 100 | 100 | 100 | 100 | 100 | --- | 99.99 | 99.99 | 99.99 | 99.99 | 99.99 | 99.99 | 99.99 | 99.99 | 99.98 | 100 | 99.99 | 100 | 99.34 | 99.34 | 99.33 | 99.34 | 99.34 | 99.34 | 99.33 | 99.36 | 99.33 | 99.34 | 99.34 | 99.34 |
| **Case II** | **GG08** | 100 | 99.99 | 99.99 | 99.99 | 99.99 | 99.99 | 99.99 | --- | 100 | 100 | 100 | 100 | 100 | 100 | 100 | 99.99 | 100 | 99.99 | 99.99 | 99.34 | 99.34 | 99.33 | 99.34 | 99.34 | 99.34 | 99.33 | 99.35 | 99.33 | 99.34 | 99.34 | 99.34 |
|  | **GG09*** | 100 | 99.99 | 99.99 | 99.99 | 99.99 | 99.99 | 99.99 | 100 | --- | 100 | 100 | 100 | 100 | 100 | 100 | 99.99 | 99.99 | 99.99 | 99.99 | 99.34 | 99.34 | 99.33 | 99.34 | 99.34 | 99.34 | 99.33 | 99.35 | 99.33 | 99.34 | 99.34 | 99.34 |
|  | **GG10** | 100 | 99.99 | 99.99 | 99.99 | 99.99 | 99.99 | 99.99 | 100 | 100 | --- | 100 | 100 | 100 | 100 | 100 | 99.99 | 100 | 99.99 | 99.99 | 99.35 | 99.35 | 99.34 | 99.35 | 99.34 | 99.34 | 99.34 | 99.36 | 99.33 | 99.34 | 99.34 | 99.35 |
|  | **GG11** | 100 | 99.99 | 99.99 | 99.99 | 99.99 | 99.99 | 99.99 | 100 | 100 | 100 | --- | 100 | 100 | 100 | 100 | 99.99 | 99.99 | 99.99 | 99.99 | 99.35 | 99.35 | 99.35 | 99.36 | 99.35 | 99.35 | 99.35 | 99.37 | 99.34 | 99.35 | 99.35 | 99.35 |
|  | **GG12** | 100 | 99.99 | 99.99 | 99.99 | 99.99 | 99.99 | 99.99 | 100 | 100 | 100 | 100 | --- | 100 | 100 | 100 | 99.99 | 100 | 99.99 | 99.99 | 99.35 | 99.35 | 99.34 | 99.35 | 99.34 | 99.34 | 99.34 | 99.36 | 99.33 | 99.34 | 99.34 | 99.35 |
|  | **GG13** | 100 | 99.99 | 99.99 | 99.99 | 99.99 | 99.99 | 99.99 | 100 | 100 | 100 | 100 | 100 | --- | 100 | 100 | 99.99 | 100 | 99.99 | 99.99 | 99.35 | 99.35 | 99.34 | 99.35 | 99.35 | 99.34 | 99.34 | 99.36 | 99.34 | 99.35 | 99.34 | 99.35 |
|  | **GG14** | 99.99 | 99.99 | 99.99 | 99.99 | 99.99 | 99.99 | 99.99 | 100 | 100 | 100 | 100 | 100 | 100 | --- | 100 | 99.99 | 100 | 99.99 | 99.99 | 99.35 | 99.34 | 99.34 | 99.35 | 99.35 | 99.34 | 99.34 | 99.36 | 99.34 | 99.34 | 99.34 | 99.35 |
|  | **GG15** | 100 | 99.99 | 99.99 | 99.99 | 99.99 | 99.99 | 99.99 | 100 | 100 | 100 | 100 | 100 | 100 | 100 | --- | 99.99 | 100 | 99.99 | 99.99 | 99.34 | 99.33 | 99.33 | 99.34 | 99.34 | 99.34 | 99.33 | 99.35 | 99.32 | 99.34 | 99.34 | 99.34 |
|  | **GG16*** | 99.98 | 99.98 | 99.98 | 99.98 | 99.99 | 99.99 | 99.98 | 99.99 | 99.99 | 99.99 | 99.99 | 99.99 | 99.99 | 99.99 | 99.99 | --- | 99.99 | 99.98 | 99.98 | 99.35 | 99.35 | 99.34 | 99.35 | 99.35 | 99.35 | 99.34 | 99.36 | 99.34 | 99.36 | 99.35 | 99.35 |
| **Case III** | **GG17*** | 100 | 100 | 100 | 100 | 100 | 100 | 100 | 100 | 99.99 | 100 | 99.99 | 100 | 100 | 100 | 100 | 99.99 | --- | 99.99 | 100 | 99.35 | 99.34 | 99.34 | 99.35 | 99.34 | 99.34 | 99.34 | 99.36 | 99.33 | 99.34 | 99.34 | 99.34 |
|  | **GG18*** | 99.99 | 99.99 | 99.99 | 99.99 | 99.99 | 99.99 | 99.99 | 99.99 | 99.99 | 99.99 | 99.99 | 99.99 | 99.99 | 99.99 | 99.99 | 99.98 | 99.99 | --- | 99.99 | 99.34 | 99.34 | 99.33 | 99.34 | 99.33 | 99.33 | 99.33 | 99.35 | 99.33 | 99.33 | 99.33 | 99.34 |
|  | **GG19** | 100 | 99.99 | 100 | 99.99 | 100 | 100 | 100 | 99.99 | 99.99 | 99.99 | 99.99 | 99.99 | 99.99 | 99.99 | 99.99 | 99.98 | 100 | 99.99 | --- | 99.35 | 99.34 | 99.34 | 99.34 | 99.34 | 99.34 | 99.33 | 99.36 | 99.33 | 99.34 | 99.34 | 99.34 |
| **Case IV** | **GG20** | 99.35 | 99.34 | 99.34 | 99.33 | 99.34 | 99.34 | 99.34 | 99.34 | 99.34 | 99.35 | 99.35 | 99.35 | 99.35 | 99.35 | 99.34 | 99.35 | 99.35 | 99.34 | 99.35 | --- | 100 | 100 | 100 | 100 | 100 | 100 | 100 | 100 | 100 | 100 | 100 |
|  | **GG21*** | 99.35 | 99.35 | 99.34 | 99.34 | 99.35 | 99.34 | 99.34 | 99.34 | 99.34 | 99.35 | 99.35 | 99.35 | 99.35 | 99.34 | 99.33 | 99.35 | 99.34 | 99.34 | 99.34 | 100 | --- | 99.99 | 100 | 100 | 100 | 100 | 100 | 99.99 | 99.99 | 100 | 100 |
|  | **GG22*** | 99.34 | 99.34 | 99.34 | 99.33 | 99.34 | 99.33 | 99.33 | 99.33 | 99.33 | 99.34 | 99.35 | 99.34 | 99.34 | 99.34 | 99.33 | 99.34 | 99.34 | 99.33 | 99.34 | 100 | 99.99 | --- | 99.99 | 99.99 | 99.99 | 100 | 100 | 99.99 | 99.99 | 100 | 100 |
|  | **GG23*** | 99.35 | 99.35 | 99.34 | 99.34 | 99.35 | 99.34 | 99.34 | 99.34 | 99.34 | 99.35 | 99.36 | 99.35 | 99.35 | 99.35 | 99.34 | 99.35 | 99.35 | 99.34 | 99.34 | 100 | 100 | 99.99 | --- | 100 | 100 | 100 | 100 | 100 | 100 | 100 | 100 |
|  | **GG24*** | 99.34 | 99.34 | 99.34 | 99.33 | 99.34 | 99.33 | 99.34 | 99.34 | 99.34 | 99.34 | 99.35 | 99.34 | 99.35 | 99.35 | 99.34 | 99.35 | 99.34 | 99.33 | 99.34 | 100 | 100 | 99.99 | 100 | --- | 100 | 100 | 100 | 100 | 100 | 100 | 100 |
| **Case V** | **GG25** | 99.34 | 99.34 | 99.33 | 99.33 | 99.34 | 99.33 | 99.34 | 99.34 | 99.34 | 99.34 | 99.35 | 99.34 | 99.34 | 99.34 | 99.34 | 99.35 | 99.34 | 99.33 | 99.34 | 100 | 100 | 99.99 | 100 | 100 | --- | 100 | 100 | 100 | 100 | 100 | 100 |
|  | **GG26** | 99.34 | 99.33 | 99.33 | 99.32 | 99.33 | 99.33 | 99.33 | 99.33 | 99.33 | 99.34 | 99.35 | 99.34 | 99.34 | 99.34 | 99.33 | 99.34 | 99.34 | 99.33 | 99.33 | 100 | 100 | 100 | 100 | 100 | 100 | --- | 100 | 100 | 100 | 100 | 100 |
|  | **GG27** | 99.36 | 99.36 | 99.35 | 99.35 | 99.36 | 99.35 | 99.36 | 99.35 | 99.35 | 99.36 | 99.37 | 99.36 | 99.36 | 99.36 | 99.35 | 99.36 | 99.36 | 99.35 | 99.36 | 100 | 100 | 100 | 100 | 100 | 100 | 100 | --- | 100 | 100 | 100 | 100 |
|  | **GG28** | 99.33 | 99.33 | 99.33 | 99.32 | 99.33 | 99.33 | 99.33 | 99.33 | 99.33 | 99.33 | 99.34 | 99.33 | 99.34 | 99.34 | 99.32 | 99.34 | 99.33 | 99.33 | 99.33 | 100 | 99.99 | 99.99 | 100 | 100 | 100 | 100 | 100 | --- | 100 | 100 | 100 |
|  | **GG29** | 99.34 | 99.34 | 99.34 | 99.33 | 99.34 | 99.34 | 99.34 | 99.34 | 99.34 | 99.34 | 99.35 | 99.34 | 99.35 | 99.34 | 99.34 | 99.36 | 99.34 | 99.33 | 99.34 | 100 | 99.99 | 99.99 | 100 | 100 | 100 | 100 | 100 | 100 | --- | 100 | 100 |
|  | **GG30** | 99.34 | 99.34 | 99.33 | 99.33 | 99.34 | 99.33 | 99.34 | 99.34 | 99.34 | 99.34 | 99.35 | 99.34 | 99.34 | 99.34 | 99.34 | 99.35 | 99.34 | 99.33 | 99.34 | 100 | 100 | 100 | 100 | 100 | 100 | 100 | 100 | 100 | 100 | --- | 100 |
|  | **GG31** | 99.34 | 99.35 | 99.34 | 99.33 | 99.34 | 99.34 | 99.34 | 99.34 | 99.34 | 99.35 | 99.35 | 99.35 | 99.35 | 99.35 | 99.34 | 99.35 | 99.34 | 99.34 | 99.34 | 100 | 100 | 100 | 100 | 100 | 100 | 100 | 100 | 100 | 100 | 100 | --- |

* Nine *S*. Bareilly strains were selected based on ANI-value and some strains with 100% ANI-value were considered identical.

**Table S6.** Total numbers of SNPs in each strain of A and B groups

| Group | Strain | Number of SNPs |
| --- | --- | --- |
| A | GG01 | 359 |
|  | GG02 | 354 |
|  | GG03 | 356 |
|  | GG04 | 355 |
|  | GG05 | 356 |
|  | GG06 | 353 |
|  | GG07 | 354 |
|  | GG08 | 355 |
|  | GG09 | 356 |
|  | GG10 | 354 |
|  | GG11 | 358 |
|  | GG12 | 355 |
|  | GG13 | 355 |
|  | GG14 | 357 |
|  | GG15 | 358 |
|  | GG16 | 359 |
|  | GG17 | 364 |
|  | GG18 | 369 |
|  | GG19 | 364 |
| B | GG20 | 18759 |
|  | GG21 | 18750 |
|  | GG22 | 18764 |
|  | GG23 | 18765 |
|  | GG24 | 18765 |
|  | GG25 | 18749 |
|  | GG26 | 18762 |
|  | GG27 | 18754 |
|  | GG28 | 18760 |
|  | GG29 | 18752 |
|  | GG30 | 18762 |
|  | GG31 | 18755 |

**TABLE S7.** Categorization of 80 *Salmonella* serotypes according to presence or absence of specific virulence factor genes of *stk, lpf,* and *tcf* gene clusters in their 411 whole genome sequences

| **Category** | ***Salmonella* serovars** | **No. of  genomes** | ***S*-specific virulence factor genes** | | | | | | | | | | | | | | | | |  |
| --- | --- | --- | --- | --- | --- | --- | --- | --- | --- | --- | --- | --- | --- | --- | --- | --- | --- | --- | --- | --- |
|  |  |  | ***stkA*** | ***stkB*** | ***stkC*** | ***stkD*** | ***stkE*** | ***stkF*** | ***stkG*** | ***lpfA*** | ***lpfB*** | ***lpfC*** | ***lpfD*** | ***lpfE*** | ***tcfA*** | ***tcfB*** | ***tcfC*** | ***tcfD*** | | |
|  | **Total** | 411 | 84 | 83 | 83 | 83 | 84 | 84 | 83 | 275 | 275 | 275 | 248 | 275 | 76 | 147 | 148 | | 88 | |
| **C1** | Anatum | 16 | 0 | 0 | 0 | 0 | 0 | 0 | 0 | 0 | 0 | 0 | 0 | 0 | 0 | 0 | 0 | | 0 | |
|  | Chester | 1 | 0 | 0 | 0 | 0 | 0 | 0 | 0 | 0 | 0 | 0 | 0 | 0 | 0 | 0 | 0 | | 0 | |
|  | Hayindogo | 1 | 0 | 0 | 0 | 0 | 0 | 0 | 0 | 0 | 0 | 0 | 0 | 0 | 0 | 0 | 0 | | 0 | |
|  | Javiana | 1 | 0 | 0 | 0 | 0 | 0 | 0 | 0 | 0 | 0 | 0 | 0 | 0 | 0 | 0 | 0 | | 0 | |
|  | Macclesfield | 1 | 0 | 0 | 0 | 0 | 0 | 0 | 0 | 0 | 0 | 0 | 0 | 0 | 0 | 0 | 0 | | 0 | |
|  | Manchester | 1 | 0 | 0 | 0 | 0 | 0 | 0 | 0 | 0 | 0 | 0 | 0 | 0 | 0 | 0 | 0 | | 0 | |
|  | Onderstepoort | 1 | 0 | 0 | 0 | 0 | 0 | 0 | 0 | 0 | 0 | 0 | 0 | 0 | 0 | 0 | 0 | | 0 | |
|  | Ouakam | 1 | 0 | 0 | 0 | 0 | 0 | 0 | 0 | 0 | 0 | 0 | 0 | 0 | 0 | 0 | 0 | | 0 | |
|  | Sloterdijk | 1 | 0 | 0 | 0 | 0 | 0 | 0 | 0 | 0 | 0 | 0 | 0 | 0 | 0 | 0 | 0 | | 0 | |
|  | Weltevreden | 5 | 0 | 0 | 0 | 0 | 0 | 0 | 0 | 0 | 0 | 0 | 0 | 0 | 0 | 0 | 0 | | 0 | |
| **C2** | Milwaukee | 1 | 1 | 1 | 1 | 1 | 1 | 1 | 1 | 0 | 0 | 0 | 0 | 0 | 0 | 0 | 0 | | 0 | |
|  | Hillingdon | 1 | 1 | 1 | 1 | 1 | 1 | 1 | 1 | 0 | 0 | 0 | 0 | 0 | 0 | 0 | 0 | | 0 | |
| **C3** | Cubana | 1 | 1 | 1 | 1 | 1 | 1 | 1 | 1 | 1 | 1 | 1 | 0 | 1 | 0 | 0 | 0 | | 0 | |
|  | Abony | 1 | 1 | 1 | 1 | 1 | 1 | 1 | 1 | 1 | 1 | 1 | 1 | 1 | 0 | 0 | 0 | | 0 | |
|  | Albany | 1 | 1 | 1 | 1 | 1 | 1 | 1 | 1 | 1 | 1 | 1 | 0 | 1 | 0 | 0 | 0 | | 0 | |
|  | Apapa | 1 | 1 | 1 | 1 | 1 | 1 | 1 | 1 | 1 | 1 | 1 | 0 | 1 | 0 | 0 | 0 | | 0 | |
|  | Stanleyville | 1 | 1 | 1 | 1 | 1 | 1 | 1 | 1 | 1 | 1 | 1 | 1 | 1 | 0 | 0 | 0 | | 0 | |
|  | Tennessee | 6 | 6 | 6 | 6 | 6 | 6 | 6 | 6 | 6 | 6 | 6 | 0 | 6 | 0 | 0 | 0 | | 0 | |
|  | Braenderup | 1 | 1 | 1 | 1 | 1 | 1 | 1 | 1 | 1 | 1 | 1 | 1 | 1 | 0 | 0 | 0 | | 0 | |
|  | Thompson | 8 | 8 | 8 | 8 | 8 | 8 | 8 | 8 | 8 | 8 | 8 | 8 | 8 | 0 | 0 | 0 | | 0 | |
|  | Bareilly  B group | 12 | 12 | 12 | 12 | 12 | 12 | 12 | 12 | 12 | 12 | 12 | 12 | 12 | 0 | 0 | 0 | | 0 | |
| **C4** | Heidelberg | 27 | 27 | 27 | 27 | 27 | 27 | 27 | 27 | 27 | 27 | 27 | 27 | 27 | 0 | 27 | 27 | | 27 | |
|  | Kentucky | 2 | 2 | 2 | 2 | 2 | 2 | 2 | 2 | 2 | 2 | 2 | 1 | 2 | 1 | 1 | 1 | | 0 | |
|  | Saintpaul | 7 | 5 | 5 | 5 | 5 | 5 | 5 | 5 | 7 | 7 | 7 | 6 | 7 | 2 | 2 | 2 | | 1 | |
|  | Senftenberg | 6 | 5 | 5 | 5 | 5 | 6 | 6 | 5 | 6 | 6 | 6 | 0 | 6 | 6 | 6 | 6 | | 0 | |
|  | Mbandaka | 2 | 1 | 1 | 1 | 1 | 1 | 1 | 1 | 1 | 1 | 1 | 0 | 1 | 1 | 1 | 1 | | 0 | |
| **C5** | Aberdeen | 1 | 0 | 0 | 0 | 0 | 0 | 0 | 0 | 1 | 1 | 1 | 1 | 1 | 1 | 1 | 1 | | 0 | |
|  | Choleraesuis | 3 | 1 | 0 | 0 | 0 | 0 | 0 | 0 | 3 | 3 | 3 | 3 | 3 | 2 | 2 | 2 | | 0 | |
|  | Infantis | 7 | 0 | 0 | 0 | 0 | 0 | 0 | 0 | 7 | 7 | 7 | 7 | 7 | 7 | 7 | 7 | | 0 | |
|  | Krefeld | 1 | 0 | 0 | 0 | 0 | 0 | 0 | 0 | 1 | 1 | 1 | 1 | 1 | 1 | 1 | 1 | | 0 | |
|  | Derby | 2 | 0 | 0 | 0 | 0 | 0 | 0 | 0 | 2 | 2 | 2 | 0 | 2 | 1 | 1 | 1 | | 0 | |
|  | Stanley | 1 | 0 | 0 | 0 | 0 | 0 | 0 | 0 | 1 | 1 | 1 | 1 | 1 | 1 | 1 | 1 | | 0 | |
|  | Wandsworth | 1 | 0 | 0 | 0 | 0 | 0 | 0 | 0 | 1 | 1 | 1 | 1 | 1 | 1 | 1 | 1 | | 0 | |
|  | Abaetetuba | 1 | 0 | 0 | 0 | 0 | 0 | 0 | 0 | 0 | 0 | 0 | 0 | 0 | 1 | 1 | 1 | | 0 | |
| **C6** | Quebec | 1 | 0 | 0 | 0 | 0 | 0 | 0 | 0 | 0 | 0 | 0 | 0 | 0 | 1 | 1 | 1 | | 0 | |
|  | Rubislaw | 1 | 0 | 0 | 0 | 0 | 0 | 0 | 0 | 0 | 0 | 0 | 0 | 0 | 1 | 1 | 1 | | 0 | |
|  | Schwarzengrund | 1 | 0 | 0 | 0 | 0 | 0 | 0 | 0 | 0 | 0 | 0 | 0 | 0 | 1 | 1 | 1 | | 0 | |
|  | Corvallis | 1 | 0 | 0 | 0 | 0 | 0 | 0 | 0 | 0 | 0 | 0 | 0 | 0 | 1 | 1 | 1 | | 0 | |
|  | Djakarta | 1 | 0 | 0 | 0 | 0 | 0 | 0 | 0 | 0 | 0 | 0 | 0 | 0 | 1 | 1 | 1 | | 0 | |
|  | Gaminara | 2 | 0 | 0 | 0 | 0 | 0 | 0 | 0 | 0 | 0 | 0 | 0 | 0 | 1 | 1 | 2 | | 0 | |
|  | Give | 2 | 0 | 0 | 0 | 0 | 0 | 0 | 0 | 0 | 0 | 0 | 0 | 0 | 2 | 2 | 2 | | 1 | |
|  | Antsalova | 1 | 0 | 0 | 0 | 0 | 0 | 0 | 0 | 0 | 0 | 0 | 0 | 0 | 1 | 1 | 1 | | 0 | |
|  | Bredeney | 2 | 0 | 0 | 0 | 0 | 0 | 0 | 0 | 0 | 0 | 0 | 0 | 0 | 2 | 2 | 2 | | 0 | |
|  | Johannesberg | 1 | 0 | 0 | 0 | 0 | 0 | 0 | 0 | 0 | 0 | 0 | 0 | 0 | 1 | 1 | 1 | | 0 | |
|  | Koessen | 1 | 0 | 0 | 0 | 0 | 0 | 0 | 0 | 0 | 0 | 0 | 0 | 0 | 1 | 1 | 1 | | 0 | |
|  | Minnesota | 2 | 0 | 0 | 0 | 0 | 0 | 0 | 0 | 0 | 0 | 0 | 0 | 0 | 2 | 2 | 2 | | 0 | |
|  | Montevideo | 7 | 0 | 0 | 0 | 0 | 0 | 0 | 0 | 0 | 0 | 0 | 0 | 0 | 7 | 7 | 7 | | 3 | |
|  | Oranienburg | 1 | 0 | 0 | 0 | 0 | 0 | 0 | 0 | 0 | 0 | 0 | 0 | 0 | 1 | 1 | 1 | | 0 | |
|  | Panama | 1 | 0 | 0 | 0 | 0 | 0 | 0 | 0 | 0 | 0 | 0 | 0 | 0 | 1 | 1 | 1 | | 0 | |
|  | Pomona | 1 | 0 | 0 | 0 | 0 | 0 | 0 | 0 | 0 | 0 | 0 | 0 | 0 | 1 | 1 | 1 | | 0 | |
|  | Poona | 2 | 0 | 0 | 0 | 0 | 0 | 0 | 0 | 0 | 0 | 0 | 0 | 0 | 2 | 2 | 2 | | 0 | |
|  | Muenster | 2 | 0 | 0 | 0 | 0 | 0 | 0 | 0 | 0 | 0 | 0 | 0 | 0 | 2 | 2 | 2 | | 0 | |
|  | India | 1 | 0 | 0 | 0 | 0 | 0 | 0 | 0 | 0 | 0 | 0 | 0 | 0 | 1 | 1 | 1 | | 0 | |
|  | Typhi | 44 | 0 | 0 | 0 | 0 | 0 | 0 | 0 | 0 | 0 | 0 | 0 | 0 | 0 | 44 | 44 | | 44 | |
|  | Bareilly  A group | 19 | 0 | 0 | 0 | 0 | 0 | 0 | 0 | 0 | 0 | 0 | 0 | 0 | 19 | 19 | 19 | | 19 | |
| **C7** | Agona | 4 | 0 | 0 | 0 | 0 | 0 | 0 | 0 | 4 | 4 | 4 | 0 | 4 | 0 | 0 | 0 | | 0 | |
|  | Bardo | 1 | 0 | 0 | 0 | 0 | 0 | 0 | 0 | 1 | 1 | 1 | 1 | 1 | 0 | 0 | 0 | | 0 | |
|  | Bergen | 1 | 0 | 0 | 0 | 0 | 0 | 0 | 0 | 1 | 1 | 1 | 0 | 1 | 0 | 0 | 0 | | 0 | |
|  | Blegdam | 1 | 0 | 0 | 0 | 0 | 0 | 0 | 0 | 1 | 1 | 1 | 1 | 1 | 0 | 0 | 0 | | 0 | |
|  | Borreze | 1 | 0 | 0 | 0 | 0 | 0 | 0 | 0 | 1 | 1 | 1 | 0 | 1 | 0 | 0 | 0 | | 0 | |
|  | Concord | 1 | 0 | 0 | 0 | 0 | 0 | 0 | 0 | 1 | 1 | 1 | 1 | 1 | 0 | 0 | 0 | | 0 | |
|  | Dublin | 2 | 0 | 0 | 0 | 0 | 0 | 0 | 0 | 2 | 2 | 2 | 2 | 2 | 0 | 0 | 0 | | 0 | |
|  | Enteritidis | 81 | 0 | 0 | 0 | 0 | 0 | 0 | 0 | 81 | 81 | 81 | 81 | 81 | 0 | 0 | 0 | | 0 | |
|  | Gallinarum | 4 | 0 | 0 | 0 | 0 | 0 | 0 | 0 | 4 | 4 | 4 | 4 | 4 | 0 | 0 | 0 | | 0 | |
|  | Hvittingfoss | 1 | 0 | 0 | 0 | 0 | 0 | 0 | 0 | 1 | 1 | 1 | 1 | 1 | 0 | 0 | 0 | | 0 | |
|  | Inverness | 1 | 0 | 0 | 0 | 0 | 0 | 0 | 0 | 1 | 1 | 1 | 1 | 1 | 0 | 0 | 0 | | 0 | |
|  | Java | 1 | 0 | 0 | 0 | 0 | 0 | 0 | 0 | 1 | 1 | 1 | 1 | 1 | 0 | 0 | 0 | | 0 | |
|  | Manhattan | 1 | 0 | 0 | 0 | 0 | 0 | 0 | 0 | 1 | 1 | 1 | 1 | 1 | 0 | 0 | 0 | | 0 | |
|  | Moscow | 1 | 0 | 0 | 0 | 0 | 0 | 0 | 0 | 1 | 1 | 1 | 1 | 1 | 0 | 0 | 0 | | 0 | |
|  | Newport | 22 | 0 | 0 | 0 | 0 | 0 | 0 | 0 | 22 | 22 | 22 | 22 | 22 | 0 | 0 | 0 | | 0 | |
|  | Nitra | 1 | 0 | 0 | 0 | 0 | 0 | 0 | 0 | 1 | 1 | 1 | 1 | 1 | 0 | 0 | 0 | | 0 | |
|  | ParatyphiC | 1 | 0 | 0 | 0 | 0 | 0 | 0 | 0 | 1 | 1 | 1 | 1 | 1 | 0 | 0 | 0 | | 0 | |
|  | Pullorum | 2 | 0 | 0 | 0 | 0 | 0 | 0 | 0 | 2 | 2 | 2 | 2 | 2 | 0 | 0 | 0 | | 0 | |
|  | Typhimurium | 54 | 0 | 0 | 0 | 0 | 0 | 0 | 0 | 54 | 54 | 54 | 54 | 54 | 0 | 0 | 0 | | 0 | |
|  | Waycross | 1 | 0 | 0 | 0 | 0 | 0 | 0 | 0 | 1 | 1 | 1 | 0 | 1 | 0 | 0 | 0 | | 0 | |
|  | Yovokome | 1 | 0 | 0 | 0 | 0 | 0 | 0 | 0 | 1 | 1 | 1 | 1 | 1 | 0 | 0 | 0 | | 0 | |
|  | 4,[5],12:i:- | 1 | 0 | 0 | 0 | 0 | 0 | 0 | 0 | 1 | 1 | 1 | 1 | 1 | 0 | 0 | 0 | | 0 | |
| **C8** | ParatyphiA | 6 | 6 | 6 | 6 | 6 | 6 | 6 | 6 | 0 | 0 | 0 | 0 | 0 | 6 | 6 | 6 | | 0 | |
|  | Cerro | 2 | 2 | 2 | 2 | 2 | 2 | 2 | 2 | 0 | 0 | 0 | 0 | 0 | 2 | 2 | 2 | | 0 | |
|  | Indiana | 1 | 1 | 1 | 1 | 1 | 1 | 1 | 1 | 0 | 0 | 0 | 0 | 0 | 1 | 1 | 1 | | 0 | |

**TABLE S8.** Antimicrobial susceptibility of *S*. Bareilly isolates.

| **Isolates** | **The interpretation of minimum inhibitory concentration** | | | | | | | | | | | | | | | | |
| --- | --- | --- | --- | --- | --- | --- | --- | --- | --- | --- | --- | --- | --- | --- | --- | --- | --- |
|  | **AM** | **AMC** | **SAM** | **CF** | **CZ** | **CTT** | **FOX** | **CTX** | **CRO** | **IPM** | **AN** | **GM** | **NA** | **CIP** | **TE** | **C** | **SXT** |
| GG-01 | S | S | S | S | S | S | S | S | S | S | S | S | S | S | S | S | S |
| GG-02 | S | S | S | S | S | S | S | S | S | S | S | S | S | S | S | S | S |
| GG-03 | S | S | S | S | S | S | S | S | S | S | S | S | S | S | S | S | S |
| GG-04 | S | S | S | S | S | S | S | S | S | S | S | S | S | S | S | S | S |
| GG-05 | S | S | S | S | S | S | S | S | S | S | S | S | S | S | S | S | S |
| GG-06 | S | S | S | S | S | S | S | S | S | S | S | S | S | S | S | S | S |
| GG-07 | S | S | S | S | S | S | S | S | S | S | S | S | S | S | S | S | S |
| GG-08 | S | S | S | S | S | S | S | S | S | S | S | S | S | S | S | S | S |
| GG-09 | S | S | S | S | S | S | S | S | S | S | S | S | S | S | S | S | S |
| GG-10 | S | S | S | S | S | S | S | S | S | S | S | S | S | S | S | S | S |
| GG-11 | S | S | S | S | S | S | S | S | S | S | S | S | S | S | S | S | S |
| GG-12 | S | S | S | S | S | S | S | S | S | S | S | S | S | S | S | S | S |
| GG-13 | S | S | S | S | S | S | S | S | S | S | S | S | S | S | S | S | S |
| GG-14 | S | S | S | S | S | S | S | S | S | S | S | S | S | S | S | S | S |
| GG-15 | S | S | S | S | S | S | S | S | S | S | S | S | S | S | S | S | S |
| GG-16 | S | S | S | S | S | S | S | S | S | S | S | S | S | S | S | S | S |
| GG-17 | S | S | S | S | S | S | S | S | S | S | S | S | S | S | S | S | S |
| GG-18 | S | S | S | S | S | S | S | S | S | S | S | S | S | S | S | S | S |
| GG-19 | S | S | S | S | S | S | S | S | S | S | S | S | S | S | S | S | S |
| GG-20 | S | S | S | S | S | S | S | S | S | S | S | S | S | S | S | S | S |
| GG-21 | S | S | S | S | S | S | S | S | S | S | S | S | S | S | S | S | S |
| GG-22 | S | S | S | S | S | S | S | S | S | S | S | S | S | S | S | S | S |
| GG-23 | S | S | S | S | S | S | S | S | S | S | S | S | S | S | S | S | S |
| GG-24 | S | S | S | S | S | S | S | S | S | S | S | S | S | S | S | S | S |
| GG-25 | S | S | S | S | S | S | S | S | S | S | S | S | S | S | S | S | S |
| GG-26 | S | S | S | S | S | S | S | S | S | S | S | S | S | S | S | S | S |
| GG-27 | S | S | S | S | S | S | S | S | S | S | S | S | S | S | S | S | S |
| GG-28 | S | S | S | S | S | S | S | S | S | S | S | S | S | S | S | S | S |
| GG-29 | S | S | S | S | S | S | S | S | S | S | S | S | S | S | S | S | S |
| GG-30 | S | S | S | S | S | S | S | S | S | S | S | S | S | S | S | S | S |
| GG-31 | S | S | S | S | S | S | S | S | S | S | S | S | S | S | S | S | S |

AM: Ampicillin, AMC: Amoxicillin/Clavulanic acid, SAM: Ampicillin/Sulbactam, CF: Cefalotin, CZ: Cefazolin, CTT: Cefotetan, FOX: Cefoxitin, CTX: Cefotaxime, CRO: Ceftriaxone, IPM: Imipenem, AN: Amikacin, GM: Gentamicin, NA: Nalidixic acid, CIP: Ciprofloxacin, TE: Tetracycline, C: Chloramphenicol, SXT: Sulfamethoxazole/Trimethoprim

**TABLE S9.** Antimicrobial susceptibility of representative *S*. Bareilly isolates.

| Isolates | Phylogenetic group | Antimicrobial resistance genes | | | | | MIC | | | |
| --- | --- | --- | --- | --- | --- | --- | --- | --- | --- | --- |
|  |  | *golS* | *mdsA* | *mdtK* | *crp* | *sdiA* | C | CIP | ERY | TE |
| GG-07 | Group A | O | O | O | O | O | 4 | ≤0.25 | >64 | ≤1 |
| GG-09 | Group A | O | O | O | O | O | 4 | ≤0.25 | >64 | ≤1 |
| GG-16 | Group A | O | O | O | O | O | 4 | ≤0.25 | >64 | ≤1 |
| GG-17 | Group A | O | O | O | O | O | 4 | ≤0.25 | >64 | ≤1 |
| GG-18 | Group A | O | O | O | O | O | 4 | ≤0.25 | >64 | ≤1 |
| GG-21 | Group B | O | O | O | O | O | 4 | ≤0.25 | >64 | ≤1 |
| GG-22 | Group B | O | O | O | O | O | 4 | ≤0.25 | >64 | ≤1 |
| GG-23 | Group B | O | O | O | O | O | 4 | ≤0.25 | >64 | ≤1 |
| GG-24 | Group B | O | O | O | O | O | 4 | ≤0.25 | >64 | ≤1 |

C: Chloramphenicol, CIP: Ciprofloxacin, ERY: Erythromycin, TE: Tetracycline

*golS*, copper efflux regulator; *mdsA*, multidrug and metal efflux complex; *mdtK*, multidrug and toxic compound extrusion transporter; *crp*, c-AMP receptor protein; *sdiA*, Cell-division regulatory protein

Breakpoint (CLSI): Chloramphenicol≥32 ug/ml, Ciprofloxacin≥4 ug/ml, Tetracycline≥16 ug/ml

**Figure legends:**

**FIGURE S1.** The SNP phylogenetic tree of 31 *S.* Bareilly strains isolated from South Korea and 67 *S.* Bareilly strains isolated from other countries. The phylogenetic tree was analyzed using the neighbor joining method and visualization of the tree was conducted using FigTree software. The sequences of the 31 S. Bareilly strains are categorized into two groups, A and B.

**FIGURE S2.** SNP analysis of 31 *S.* Bareilly strains isolated from South Korea. (A) SNP variant sequence visualization of 31 *S*. Bareilly, comparing to *S.* Bareilly FC745 as a reference strain. The left side of the figure represents group name (A or B) and strain name of 31 *S.* Bareilly. Grey color indicates the match region to the reference genome of FC745 and cyan color indicates the variant sequence region. Scale bar is kb. (B) Diagram of core SNPs in A group, B group, and A/B group.

**FIGURE S3.** ANI analysis of various *Salmonella* serotypes in C3 and C6.

**FIGURE S4.** Physiological characterization of *S*. Bareilly outbreak strains. (A) Adhesion and (B) Invasion capacity. Caco-2 cells were infected with *S*. Bareilly and *S*. Typhimurium 14028 grown for 1 h at 37 °C (MOI 10). After cells were lysed, invaded and adhered bacteria were enumerated by plating. Each bar shows the mean ± standard error of the mean (SEM) from replicates of three independent experiments.

**FIGURE S1.**


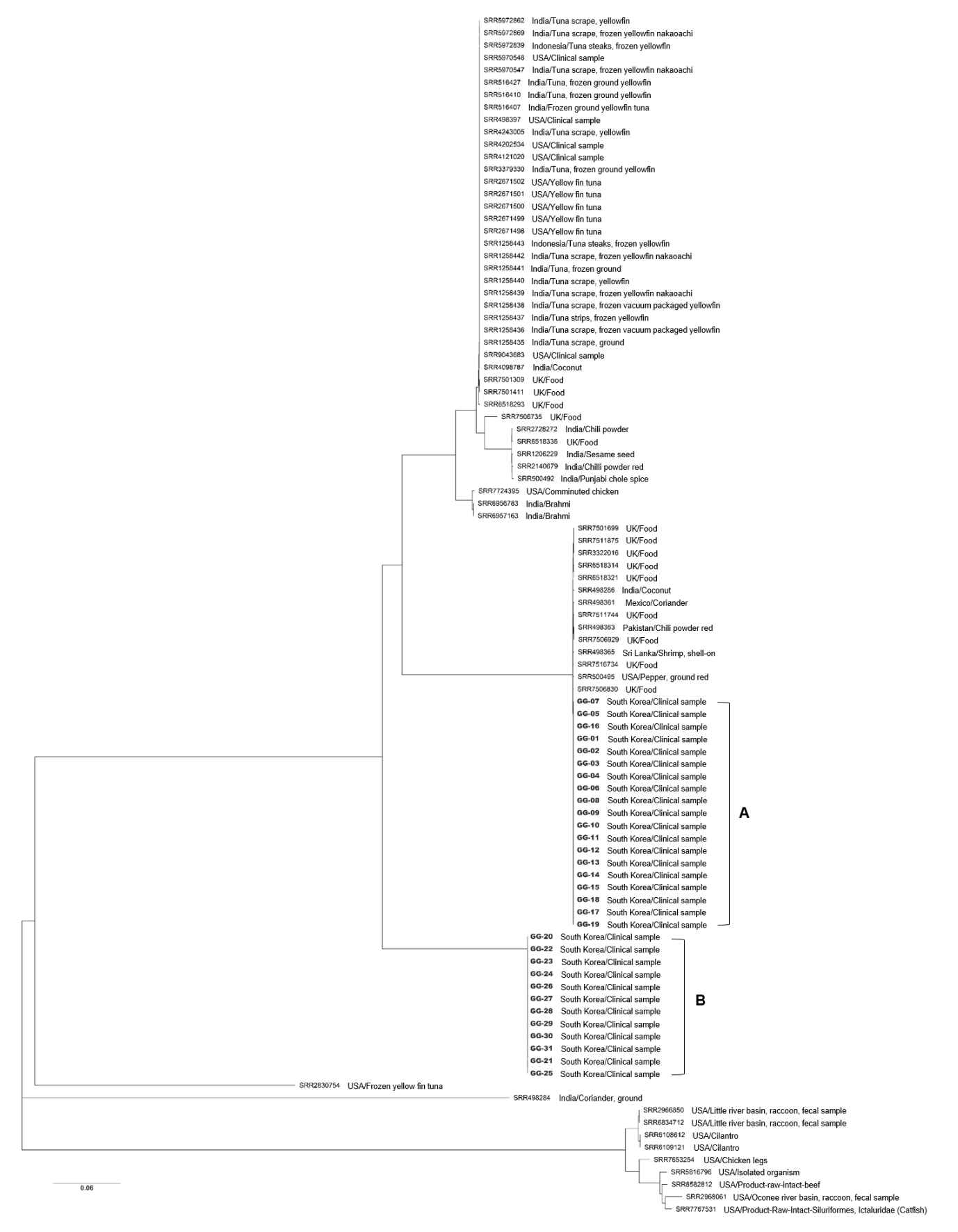


**FIGURE S2.**


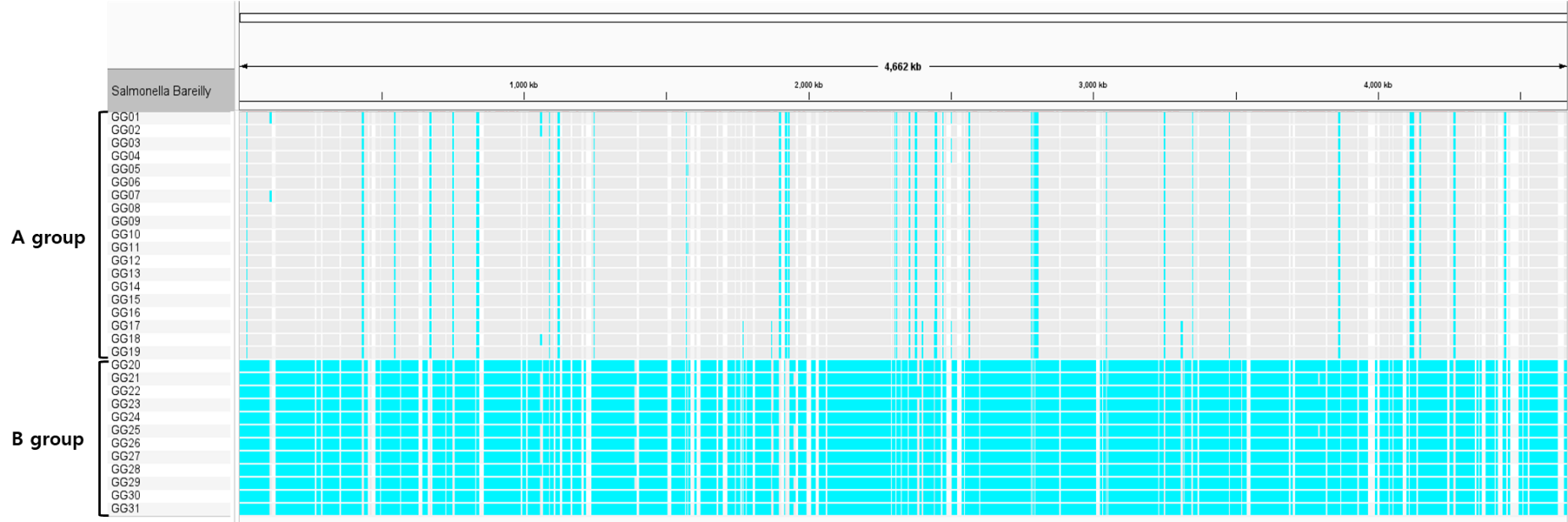


**A**

**
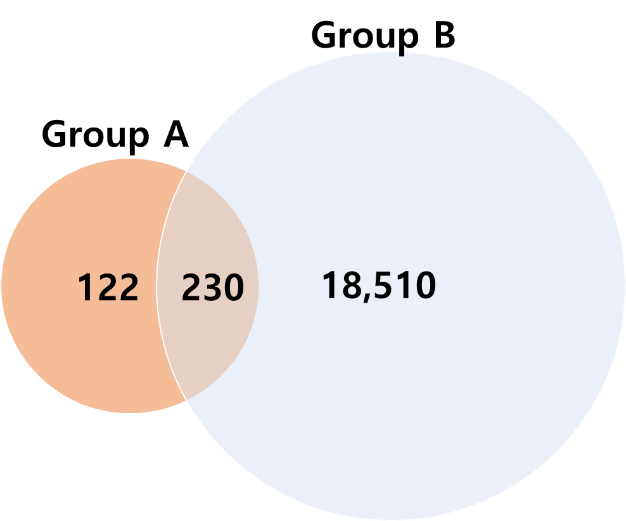
B**

**FIGURE S3.**

**
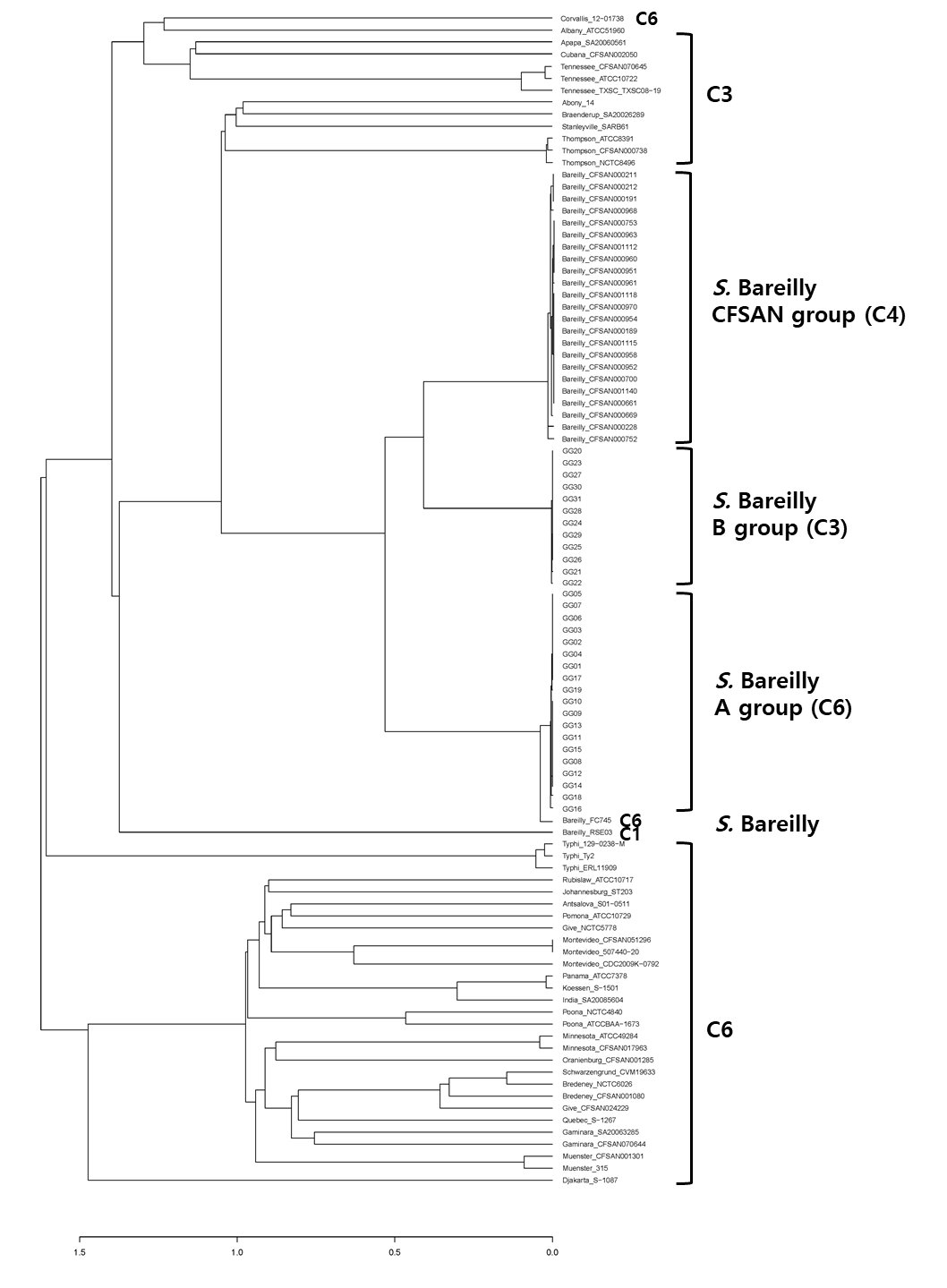
**

**FIGURE S4.**


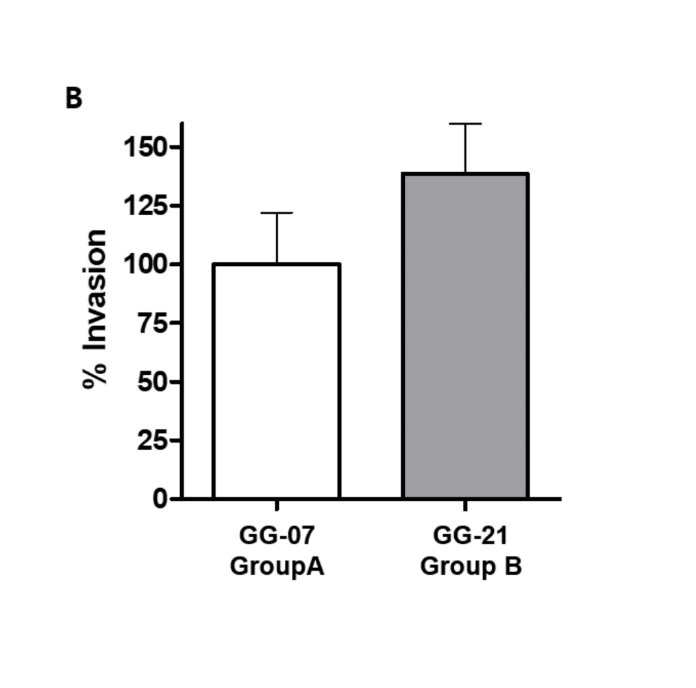

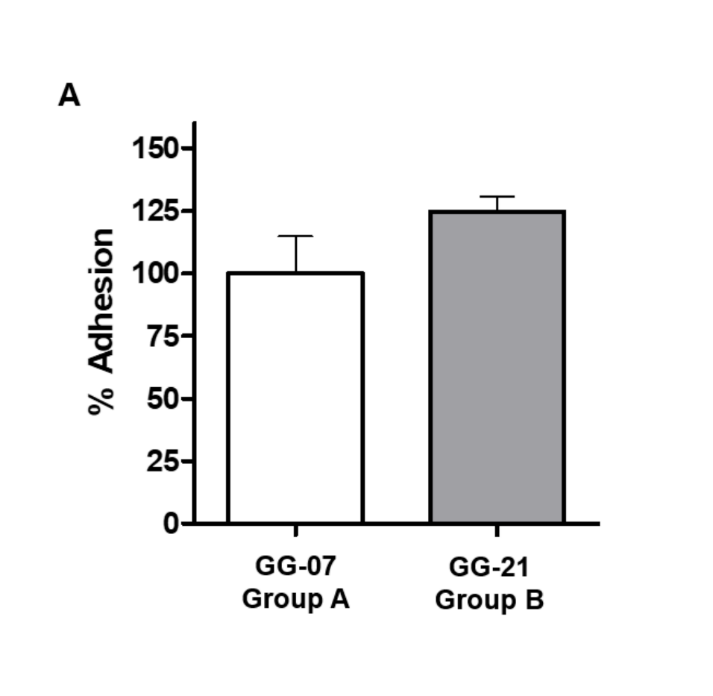

Supplement: Supplementary file 1 [file Data_Sheet_1.docx]
